# Nano‐Armed *Limosilactobacillus reuteri* for Enhanced Photo‐Immunotherapy and Microbiota Tryptophan Metabolism against Colorectal Cancer

**DOI:** 10.1002/advs.202410011

**Published:** 2024-12-30

**Authors:** Haiting Xu, Yajun Wang, Ga Liu, Zhenhua Zhu, Mohammad‐Ali Shahbazi, Rui L. Reis, Subhas C. Kundu, Xiaoxiao Shi, Menghang Zu, Bo Xiao

**Affiliations:** ^1^ State Key Laboratory of Resource Insects College of Sericulture Textile, and Biomass Sciences Southwest University Chongqing 400715 China; ^2^ Department of Pharmacy Personalized Drug Therapy Key Laboratory of Sichuan Province Sichuan Academy of Medical Sciences & Sichuan Provincial People's Hospital School of Medicine University of Electronic Science and Technology Chengdu 610054 China; ^3^ Department of Gastroenterology The First Affiliated Hospital of Nanchang University Nanchang 330006 China; ^4^ Department of Biomedical Engineering University Medical Center Groningen University of Groningen Antonius Deusinglaan 1 Groningen 9713 AV Netherlands; ^5^ W.J. Kolff Institute for Biomedical Engineering and Materials Science University of Groningen Antonius Deusinglaan 1 Groningen 9713 AV Netherlands; ^6^ 3Bs Research Group I3Bs — Research Institute on Biomaterials Biodegradables and Biomimetics University of Minho Headquarters of the European Institute of Excellence on Tissue Engineering and Regenerative Medicine AvePark, Barco Guimarães 4805‐017 Portugal; ^7^ ICVS/3B's‐PT Government Associate Laboratory Braga Guimarães 4800‐058 Portugal

**Keywords:** oral administration, colorectal cancer, engineered bacteria, phototherapy, immunotherapy

## Abstract

Despite being a groundbreaking approach to treating colorectal cancer (CRC), the efficacy of immunotherapy is significantly compromised by the immunosuppressive tumor microenvironment and dysbiotic intestinal microbiota. Here, leveraging the superior carrying capacity and innate immunity‐stimulating property of living bacteria, a nanomedicine‐engineered bacterium, LR‐S‐CD/CpG@LNP, with optical responsiveness, immune‐stimulating activity, and the ability to regulate microbiota metabolome is developed. Immunoadjuvant (CpG) and carbon dot (CD) co‐loaded plant lipid nanoparticles (CD/CpG@LNPs) are constructed and conjugated to the surface of *Limosilactobacillus reuteri* (LR) via reactive oxygen species (ROS)‐responsive linkers. The inherent photothermal and photodynamic properties of oral CD/CpG@LNPs induce in situ cytotoxic ROS generation and immunogenic cell death of colorectal tumor cells. The generated neoantigens and the released CpG function as a potent in situ vaccine that stimulates the maturation of immature dendritic cells. The mature dendritic cells and metabolites secreted by LR subsequently facilitated the tumor infiltration of cytotoxic T lymphocytes to eradicate colorectal tumors. The further in vivo results demonstrate that the photo‐immunotherapy and intestinal microbial metabolite regulation of LR‐S‐CD/CpG@LNPs collectively suppressed the growth of orthotopic colorectal tumors and their liver metastases, presenting a promising avenue for synergistic treatment of CRC via the oral route.

## Graphical abstract

A nanomedicine‐armed probiotic is constructed to promote tumor accumulation of therapeutics, regulate the intestinal microbiota and their metabolites, and activate systemic anti‐tumor immune responses against colorectal cancer and liver metastasis.

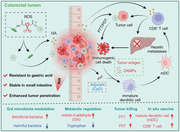

## 1 Introduction

Bacterium‐based tumor immunotherapy has a long history dating back to the late 19^th^ century and remains a prominent area of research today.^[^ ^1^ ^]^ The efficacy of bacterial therapy is attributed to the inherent tumor‐colonizing capability exhibited by anaerobic or facultative anaerobic bacteria (e.g., *Salmonella*, *Escherichia*, and *Listeria*) as well as their versatile delivering function via surface modifications.^[^ ^2^, ^3^, ^4^, ^5^, ^6^, ^7^ ^]^ Recently, probiotic strains of *Lactobacillus* have attracted great interest due to their ability to modulate immune responses and intestinal microbiota.^[^ ^8^, ^9^, ^10^, ^11^ ^]^ Among these strains, *Limosilactobacillus reuteri* (LR) has been demonstrated to secrete indole‐3‐aldehyde (I3A), a dietary tryptophan catabolite that functions as an agonist for the aryl hydrocarbon receptor (AHR).^[^ ^12^, ^13^, ^14^ ^]^ I3A also stimulates the production of interferon‐*γ* (IFN‐*γ*) by CD8^+^ T cells, thereby augmenting the anti‐tumor immune responses.^[^ ^13^, ^15^ ^]^

Colorectal cancer (CRC) is a major health concern across the globe, accounting for 10% of all cancer cases each year.^[^ ^16^ ^]^ Its hepatic metastases are the primary cause of mortality among CRC patients.^[^ ^17^ ^]^ Conventional therapeutic modalities, including surgery, chemotherapy, and radiotherapy, constitute the cornerstone of CRC management.^[^ ^18^ ^]^ However, despite advancements in these therapeutic approaches, the incidence and mortality rates of CRC continue to escalate at an alarming pace. In the past decade, immunotherapy has generated considerable enthusiasm owing to its capacity to elicit enduring and sustainable responses in solid tumors that were previously refractory to treat.^[^ ^19^, ^20^ ^]^ The immune microenvironment of colorectal tumors plays a critical role in the disease's biology, rendering it a key target for therapeutic intervention.^[^ ^21^ ^]^ Furthermore, increasing evidence supports the significant contribution of gut microbiota and its metabolites to the immune response associated with CRC therapies.^[^ ^13^, ^22^, ^23^, ^24^ ^]^ However, the immunosuppressive tumor microenvironment (TME) of CRC has hindered the activation of innate immune cells and impedes adaptive immune cells from exerting their antitumor activities, leading to inadequate responses of tumors to immunotherapies.^[^ ^25^, ^26^ ^]^ Phototherapies, encompassing both photothermal therapy (PTT) and photodynamic therapy (PDT), not only exhibit precise and highly efficient performance in tumor elimination, but also possess the potential to convert immune‐silent “cold” tumors into immune‐inflamed “hot” tumors by inducing immunogenic cell death (ICD), releasing tumor‐associated antigens (TAAs) and damage‐associated molecular patterns (DAMPs).^[^ ^27^, ^28^, ^29^, ^30^ ^]^ The “hot” tumors show an enhanced response towards different immunotherapies, such as immunoadjuvants and bacteria‐mediated immunotherapy.^[^ ^31^, ^32^, ^33^ ^]^ Oral administration is the most common and practical route for treating gastrointestinal diseases due to convenience, patient compliance, and direct drug delivery.^[^ ^34^ ^]^ Inspired by the distinctive innate immune capacities of native bacteria, it is hypothesized that oral probiotic bacteria can outcompete pathogenic microorganisms within the gut and rebalance the intestinal population.^[^ ^35^, ^36^ ^]^

**Figure 1:**
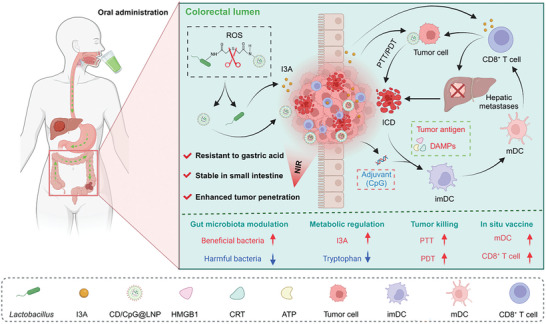
Schematic illustration of oral LR‐S‐CD/CpG@LNPs to achieve tumor accumulation, in situ vaccination, and activation of systemic antitumor immune responses against CRC. After oral administration, nanotherapeutics effectively pass through the upper GIT and accumulate in the deep orthotopic tumor tissues under NIR irradiation. Subsequently, CD/CpG@LNPs induce ICD of tumor cells via PTT/PDT and release abundant autoneoantigens. Neoantigens and CpG are employed in concert to facilitate DC maturation and promote the infiltration of CTLs into the colorectal tumor tissues. Moreover, *Lactobacillus* enhances the antitumor immune responses by upregulation the level of I3A through the tryptophan metabolic pathway. The synergistic treatment modality of PTT/PDT, in situ vaccination, and I3A not only suppresses orthotopic tumors, but also activates systematic antitumor immunity against distant tumors, increases the abundance of gut beneficial bacteria, and decreases the abundance of harmful bacteria.

Here, carbon dots (CDs) and immunoadjuvant‐intensified LR probiotic were combined (denoted as LR‐S‐CD/CpG@LNPs) to achieve efficient photo‐immunotherapy and microbial metabolite regulation against metastatic CRC. Considering the aerobic nature of LR, a reactive oxygen species (ROS)‐cleavable linker was employed to connect LR with CDs and CpG‐encapsulated mulberry leaf lipid (MLL) nanoparticles (LNPs), which enabled the prolonged retention of CD/CpG@LNPs in the colorectal tumors.^[^ ^37^ ^]^ After oral administration, LR‐S‐CD/CpG@LNPs preferentially accumulated in the colorectal regions rich in ROS, causing the linkers to be broken and facilitating the accumulation of CD/CpG@LNPs into the colorectal tumors. Upon near‐infrared (NIR) irradiation, the temperature and cytotoxic ROS levels in the tumor area sharply increased, resulting in the rupture and demise of tumor cells from ICD. Subsequently, the released TAAs from tumor cells and CpG molecules from CD/CpG@LNPs formed in situ vaccines, which promoted the recruitment and maturation of dendritic cells (DCs). Meanwhile, LR catalyzed the metabolism of tryptophan, leading to the production of I3A, a key metabolite that amplified antitumor immune responses. Additionally, LR administration induced specific alterations in the composition of gut microbiota, promoting an increase in the abundance of beneficial bacteria, such as *Lactobacillus* and *Alistipes*, while concurrently reducing the prevalence of harmful bacteria, like *Enterobacteriaceae*. The effectiveness of this bacteria‐based anti‐tumor system in eliminating colorectal tumors has also been validated by in vivo studies, showcasing its promising potential for treating CRC and its liver metastases (**Figure** **1**).

## 2 Results and Discussion

### 2.1 Fabrication and Physicochemical Characterization of CD/CpG@LNPs and LR‐S‐CD/CpG@LNPs

**Figure 2:**
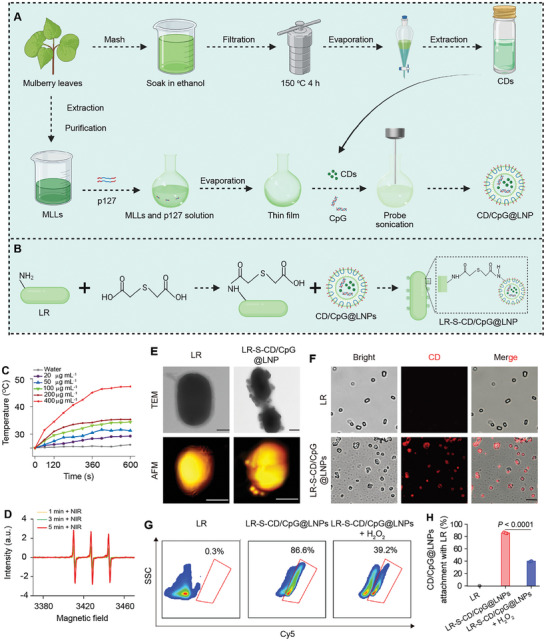
Preparation and physicochemical characterization of LR‐S‐CD/CpG@LNPs. Preparation diagram of A) CDs and CD/CpG@LNPs and B) LR‐S‐CD/CpG@LNPs. Insets (A,B) were created with BioRender.com. C) Temperature variations of water and CD suspensions with different CD concentrations as a function of irradiation time with a 660 nm laser (1.0 W cm^−2^). D) ESR spectra of ^1^O_2_ generation of CD/CpG@LNPs upon NIR irradiation with a 660 nm laser (1.0 W cm^−2^) for different time intervals. E) TEM and AFM images of LR and LR‐S‐CD/CpG@LNPs. Scale bar: 500 nm. F) CLSM images of LR and LR‐S‐CD/CpG@LNPs. The red channel shows CDs. G) FCM analysis of LR‐S‐CD/CpG@LNPs in the absence and presence of H_2_O_2_ treatment and H) the corresponding quantitative results (n = 3 independent experiments; *p < 0.05, **p < 0.01, ***p < 0.001, and ****p < 0.0001 by one‐way ANOVA with Tukey's multiple comparison test).

MLL‐based LNPs encapsulating CDs and CpG (CD/CpG@LNPs) were constructed using a thin film hydration technique (**Figure** **2**A), which were further conjugated to the surface of LR (Figure 2B). Initially, CDs were synthesized through a solvothermal reaction utilizing mulberry leaves as an exclusive source of carbonaceous precursors. The obtained CDs possessed a uniform spherical nanostructure with a mean hydrodynamic diameter of 2.2 nm and a polydispersity index (PDI) of 0.21, as determined by transmission electron microscopy (TEM) and dynamic light scattering (DLS), respectively (Figure S1A,B, Supporting Information). The prominent NIR absorption peak observed at 660 nm indicated the potential of CDs to perform phototherapy (Figure S1C, Supporting Information).

It is known that oral therapeutics are prone to be degraded in the gastrointestinal tract (GIT), necessitating protective delivery systems. Our recent studies have demonstrated that LNPs made of MLLs and an FDA‐approved polymer, Pluronic 127 (p127), had excellent biosafety and could stably traverse the GIT for targeted drug delivery to the diseased colorectal tissues.^[^ ^38^ ^]^ By mixing MLL, p127, photosensitizer (CD), and immunoadjuvant (CpG), CD/CpG@LNPs were produced. The DLS data revealed that the CD/CpG@LNPs possessed a hydrodynamic diameter of 188.2 nm and showed a narrow size distribution (PDI = 0.27). They remained virtually unaltered upon exposure to various simulated gastrointestinal fluids (pH 2.0, 6.8, and 6.0), demonstrating their favorable stability in the GIT (Figure S1D,E, Supporting Information). The representative TEM and atomic force microscope (AFM) images depicted a spherical morphology of CD/CpG@LNPs, with a diameter of 133.5 nm (Figure S1F,G, Supporting Information). The encapsulation efficiency of CpG in CD/CpG@LNPs was determined to be 68.2% using an agarose gel electrophoresis assay (Figure S1H, Supporting Information). The successful and highly efficient encapsulation of CpG offers the potential for in vivo immunoregulation.

Encouraged by the specific spectral band (660 nm) in the NIR region of CDs, we inspected the optical properties of CD/CpG@LNPs under NIR. The photothermal effect of CD/CpG@LNPs was in proportion to their concentrations, as well as to the radiant intensity and laser exposure duration, demonstrating the potential for precise temperature control capability of these LNPs (Figures 2C and S1I, Supporting Information). The CD/CpG@LNPs also displayed good photothermal cycling stability and a conversion efficiency of 33.2% (Figure S2A,B, Supporting Information). It is worth noting that a satisfactory NIR photothermal effect on orthotopic CRC mice receiving oral CD/CpG@LNPs was obtained (Figure S3A,B, Supporting Information). In addition to its exceptional photothermal performance, the photodynamic property of CD/CpG@LNPs in ROS generation was likewise excellent, as evidenced by electron spin resonance (ESR) analysis (Figure 2D). High ROS levels were found in the colorectal tumors of CRC patients.^[^ ^39^ ^]^ Interestingly, a single thioether linkage exhibits specific responsiveness to ROS, undergoing cleavage within the ROS‐rich microenvironment to facilitate rapid drug release.^[^ ^32^ ^]^ To further facilitate the tumor accumulation of CD/CpG@LNPs, single thioether bonds as linkers were employed to conjugate CD/CpG@LNPs onto the surface of LR (Figure 2B). TEM and AFM images uncovered their successful chemical conjugations, as indicated by the presence of spherical CD/CpG@LNP nanostructures attached to the LR surface in the LR‐S‐CD/CpG@LNPs, in contrast to the non‐conjugated LR cells, which exhibited a typical rod shape with well‐defined smooth edges (Figure 2E). The DLS measurements revealed an increase in particle size and a decrease in zeta potential following conjugation, confirming the successful construction of LR‐S‐CD/CpG@LNPs (Figure S4A,B, Supporting Information). The confocal laser scanning microscopy (CLSM) showed a robust red fluorescence of CDs on the LR surface, again illustrating the successful conjugation of CD/CpG@LNPs to the LR (Figure 2F).

LR is an aerobe, which may impede infiltration into the hypoxic tumor region. It is thus imperative to ensure the release of CD/CpG@LNPs from LR to the tumor tissues. The flow cytometric (FCM) analysis revealed a significant decrease in fluorescent intensity of LR‐S‐CD/CpG@LNPs when exposed to hydrogen peroxide (H_2_O_2_), indicating good detachment of the CD/CpG@LNPs from LR in the colorectal TME (Figure 2G,H). To optimize the anchoring efficiency of CD/CpG@LNPs on the LR surface, we adjusted the ratios between CD/CpG@LNPs and LR. It was discovered that when the CD level reached 400 µg, the attachment of CD/CpG@LNPs (10^8^ CFU LR) achieved saturation with a conjugation efficiency of 67.5% (Figure S5A, Supporting Information). Notably, the growth tendencies of both LR and LR‐S‐CD/CpG@LNPs in the deMan Rogosa Sharpe (MRS) medium were found to be similar, confirming the negligible impact of CD/CpG@LNPs on the growth of LR (Figure S5B, Supporting Information). Moreover, the bonding of CD/CpG@LNPs on the LR surface had minimal influence on the photothermal properties of CDs (Figure S5C, Supporting Information). The activity of LR in both non‐conjugated and LR‐S‐CD/CpG@LNP‐conjugated states remained unchanged in the simulated GIT environment (Figure S5D–F, Supporting Information).

### 2.2 In Vitro Cellular Uptake, Antitumor Effects, and Antitumor Immune Activation of CD/CpG@LNPs

**Figure 3:**
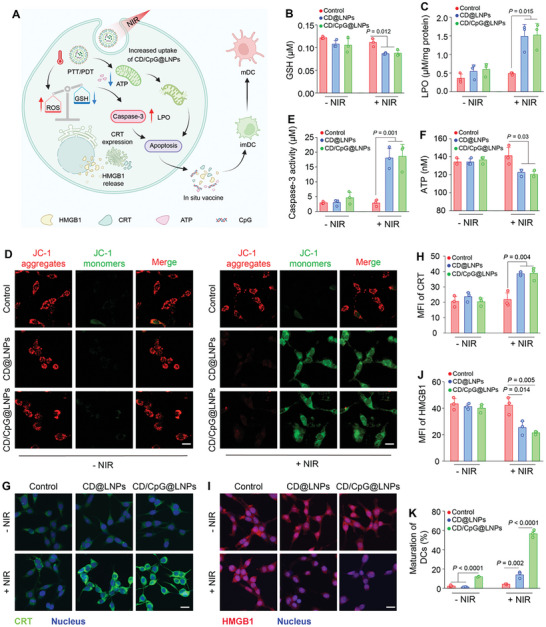
In vitro pro‐apoptosis, ICD, and DC pro‐maturation effects of CD/CpG@LNPs. A) Schematic illustration of the therapeutic mechanism of CD/CpG@LNPs based on phototherapy‐induced apoptosis and ICD, as well as CpG‐regulated DC maturation. Inset (A) was created with BioRender.com. B) The contents of GSH in CT‐26 cells incubated with CD@LNPs and CD/CpG@LNPs at a CD concentration of 2 µg mL^−1^ with or without NIR irradiation (660 nm, 0.5 W cm^−2^) for 3 min. C) Quantifying LPO in CT‐26 cells incubated with CD@LNPs and CD/CpG@LNPs at a CD concentration of 2 µg mL^−1^ with or without NIR irradiation. D) CLSM images of mitochondrial membrane potential changes in CT‐26 cells after different treatments (scare bar: 20 µm). E) Relative enzymatic activities of caspase‐3 in CT‐26 cells incubated with CD@LNPs and CD/CpG@LNPs at a CD concentration of 2 µg mL^−1^ with or without NIR irradiation. F) Intracellular ATP levels of CT‐26 cells after different treatments. G) CLSM images showing the CRT profiles of CT‐26 cells and H) the corresponding mean fluorescence intensities (MFIs). I) CLSM images showing the HMGB1 profiles of CT‐26 cells and J) the corresponding MFI (scare bar: 20 µm). K) FCM quantification of DC maturation after different treatments (n = 3 independent experiments; *p < 0.05, **p < 0.01, ***p < 0.001, and ****p < 0.0001 by one‐way ANOVA with Tukey's multiple comparison test).

Prior to estimating the phototherapeutic effect of CD/CpG@LNPs against CT‐26 cells, we evaluated their cellular uptake efficiencies. The CLSM images showed that DiO‐LNPs successfully entered the cytoplasm of CT‐26 cells, and the intracellular level of LNPs was increased after NIR irradiation as demonstrated by quantitative FCM results (Figure S6A,B, Supporting Information). The methylthiazolyldiphenyl‐tetrazolium bromide (MTT) assay revealed that upon exposure to NIR irradiation, both CD@LNPs and CD/CpG@LNPs had pronounced in vitro anti‐tumor capacities, as seen from the reduction in viability of CT‐26 cells to below 13.3% (Figure S6C,D, Supporting Information). The live/dead cell staining assay yielded similar results (Figure S7A, Supporting Information). The remarkable eradication of tumor cells could not be divorced from NIR‐induced overproduction of ROS and depletion of glutathione (GSH) (**Figure** **3**B; Figure S7B,C, Supporting Information). These phenomena can result in excessive accumulation of lipid peroxidation (LPO) within cells, thereby contributing to the antitumor efficacy of immunotherapy.^[^ ^40^, ^41^ ^]^ As expected, we found that NIR irradiation significantly induced CD@LNPs and CD/CpG@LNPs to generate 3.4‐fold higher LPO levels compared to the other groups (Figure 3C). In view of the probable damage to the mitochondria caused by the phototherapy, we measured the mitochondrial membrane potential of CT‐26 cells post‐treatment. The hypothesis was confirmed by the absence of red signals and largely emerged green fluorescence in the CD@LNP (+ NIR) and CD/CpG@LNP (+ NIR) groups (Figure 3D). Consequently, mitochondrial destruction induced the upregulation of caspase‐3 by 6.1‐fold in cells treated with CD@LNPs and CD/CpG@LNPs upon NIR irradiation, ultimately leading to caspase‐3‐dependent apoptosis (Figure 3E).

Since phototherapy possesses the capability of triggering ICD,^[^ ^42^ ^]^ the levels of DAMPs characteristic of ICD of tumor cells, including calreticulin (CRT), high‐mobility group box 1 protein (HMGB1), and adenosine triphosphate (ATP), were determined. Only under 660 nm laser irradiation, CD@LNPs, and CD/CpG@LNPs caused apparent intracellular ATP secretion (Figure 3F), surface exposure of CRT (Figure 3G,H), and extracellular release of HMGB1 (Figure 3I,J) in CT‐26 cells. The ICD of tumor cells can enhance the maturation of DCs and facilitate their efficient antigen presentation to cytotoxic T lymphocytes (CTLs). As shown in Figure 3K, the DC maturation percentages of two NIR‐involved groups were higher than their non‐irradiated counterparts. Particularly, the presence of CpG in CD/CpG@LNPs greatly enhanced DC maturation, achieving up to 56.6% maturation upon NIR irradiation. This result can be attributed to the release of CpG from CD/CpG@LNPs and activation of the Toll‐like receptor 9 (TLR9) signaling pathway by CpG, which conduces to regulating DC maturation, stimulating the production of pro‐inflammatory cytokines and priming naive T cells for acquired anti‐tumor immunity^[^ ^43^, ^44^, ^45^ ^]^ (Figure 3A). The above findings suggest that the coexistence of CDs and CpG in CD/CpG@LNPs can induce ICD of tumor cells, while promoting DC maturation, positioning them as a promising candidate for photo‐immunotherapy against CRC.

### 2.3 In Vivo Bio‐Distribution of CD/CpG@LNPs and LR‐S‐CD/CpG@LNPs

**Figure 4:**
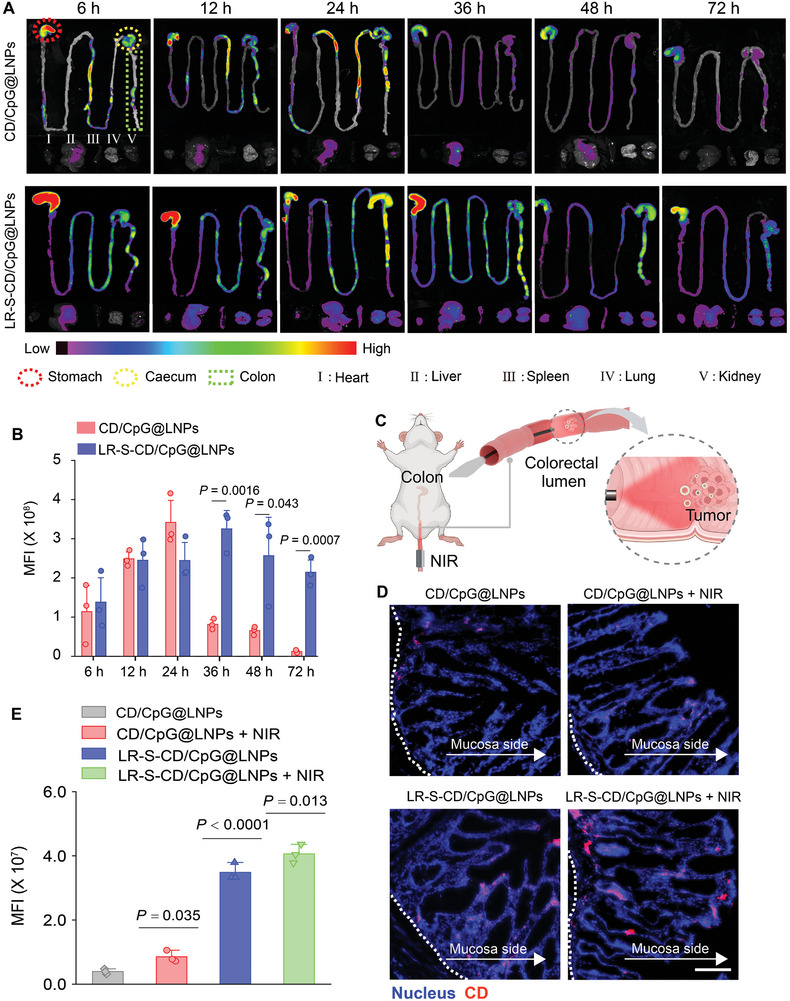
Tumor accumulation and in vivo distribution of CD/CpG@LNPs and LR‐S‐CD/CpG@LNPs with or without NIR irradiation after oral administration. A) Ex vivo fluorescence imaging of the GITs from mice after oral administration of CD/CpG@LNPs and LR‐S‐ CD/CpG@LNPs at 6, 12, 24, 48, and 72 h. B) Following oral administration of CD/CpG@LNPs or LR‐S‐CD/CpG@LNPs at 6, 12, 24, 48, and 72 hours, the corresponding MFIs of the colon were measured. C) Function diagram of the NIR optical fiber for CRC treatment. D) Frozen section imaging of colons from CRC mice receiving oral administration of CD/CpG@LNPs and LR‐S‐CD/CpG@LNPs for 36 h with or without NIR (scare bar: 100 µm). E) MFI of CDs in the colon tissues from various groups (n = 3 independent experiments; *p < 0.05, **p < 0.01, ***p < 0.001, and ****p < 0.0001 by one‐way ANOVA with Tukey's multiple comparison test).

Given the difficulty of aerobic bacteria to colonize the hypoxic TME with abundant ROS, ROS‐responsive linkers were employed to improve the release, penetration, and accumulation of CD/CpG@LNPs in the colorectal tumors. The *ex vivo* biodistribution analysis suggested that following oral administration, compared to the rapid clearance of CD/CpG@LNPs alone, LR enabled their prolonged retention in the colon for up to 72 h (**Figure** **4**A,B). To improve the therapeutic potential of NIR in treating colonic diseases, we designed a specialized NIR fiber (Figure 4C) that can be effortlessly inserted into the colonic lumen for NIR light emission. The cryosection images revealed that the LR‐S‐CD/CpG@LNP group exhibited brighter red fluorescence signals in the colorectal tumor tissues at 36 h compared to the CD/CpG@LNP group. In addition, we found that large quantities of CD/CpG@LNPs were present in the tumor tissues with NIR irradiation (Figure 4D,E). These observations indicate that LR‐S‐CD/CpG@LNPs can effectively release CD/CpG@LNPs in response to the TME characterized by high ROS concentrations, resulting in the accumulation and retention of released CD/CpG@LNPs within the tumor tissues. We also found that applying NIR irradiation increased the cumulative amounts of NPs in the tumor tissues.

### 2.4 In Vivo Biosafety Evaluation of Oral LR‐S‐CD/CpG@LNPs Plus NIR

Excellent biosafety is essential for the successful clinical translation of LR‐S‐CD/CpG@LNPs in combination with NIR irradiation.^[^ ^46^ ^]^ To assess the in vivo biosafety of LR‐S‐CD/CpG@LNPs (+ NIR), Balb/c mice were orally administered a total of three doses of LR‐S‐CD/CpG@LNPs (CD, 3 mg kg^−1^; CpG, 16 µg; 200 µL). Strikingly, no significant difference in body weight was observed in the LR‐S‐CD/CpG@LNP (+ NIR)‐treated mice, compared to the water group throughout the experimental period (Figure S8A, Supporting Information). After completion of the treatments, there were no significant differences in organ index or colon length between the water group and the LR‐S‐CD/CpG@LNP (+ NIR) group (Figure S8B,C, Supporting Information). Comprehensive blood analysis revealed no notable variations in white blood cells, granulocytes, and red blood cells, all of which remained within normal ranges (Table S1, Supporting Information). To evaluate the potential hepatotoxicity and nephrotoxicity, serum biomarkers, including alanine aminotransferase (ALT), alkaline phosphatase (AKP), creatinine (CRE), and blood urea nitrogen (BUN), were analyzed. The LR‐S‐CD/CpG@LNP (+ NIR) treatment did not cause any abnormal fluctuations of these markers (Figure S8D, Supporting Information). These findings collectively demonstrate the excellent safety of LR‐S‐CD/CpG@LNPs plus NIR irradiation.

### 2.5 In Vivo Antitumor Effects of Oral CD/CpG@LNPs and LR‐S‐CD/CpG@LNPs against Orthotopic CRC

**Figure 5:**
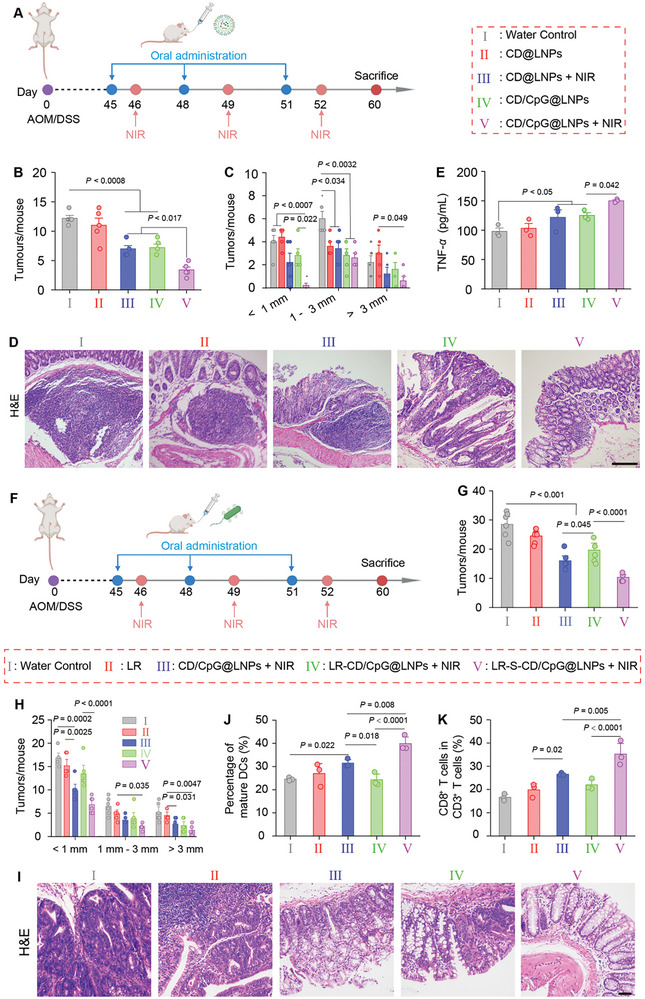
In vivo therapeutic outcomes of various modalities against orthotopic CRC. A) Treatment protocol of different modalities against orthotopic colorectal tumors. B) Total tumor numbers and C) numbers of different‐size tumors per mouse (n = 5 biologically independent experiments; Data were analyzed by one‐way ANOVA with Tukey's multiple comparison test). D) H&E staining of colorectal tumor sections from various mouse groups (scale bar = 100 µm). E) Levels of serum TNF‐*α* from various mouse groups (n = 3 biologically independent experiments; *p < 0.05, **p < 0.01, ***p < 0.001, and ****p < 0.0001 by one‐way ANOVA with Tukey's multiple comparison test). F) Treatment protocol of LR‐S‐CD/CpG@LNPs (+ NIR) against orthotopic CRC. G) Total tumor numbers and H) numbers of different‐size tumors per mouse (n = 6 biologically independent experiments; *p < 0.05, **p < 0.01, ***p < 0.001, and ****p < 0.0001 by one‐way ANOVA with Tukey's multiple comparison test). I) H&E staining of colorectal tumor sections from various mouse groups (scale bar = 100 µm). J) Percentages of mDCs in the tumor‐draining mesenteric lymph nodes from various mouse groups. K) Percentages of CD8^+^ T cells in the spleens from various mouse groups (n = 3 biologically independent experiments; *p < 0.05, **p < 0.01, ***p < 0.001, and ****p < 0.0001 by one‐way ANOVA with Tukey's multiple comparison test).

The azoxymethane (AOM)/dextran sodium sulfate (DSS)‐induced mouse model effectively simulates human colorectal cancer.^[^ ^47^ ^]^ Thus, we evaluated the antitumor efficacy of CD/CpG@LNPs in a mouse model bearing orthotopic colorectal tumors, as illustrated in **Figure** **5**A, CD@LNPs without NIR irradiation could not retard the growth of colorectal tumors, similar to the water control group (Figure 5B,C; Figure S9, Supporting Information). However, with the introduction of NIR irradiation or CpG, the numbers and sizes of colorectal tumors were decreased. Strikingly, the inhibitory effect was further improved when subjected to the simultaneous effects of NIR irradiation and CpG, resulting from the combined effects of phototherapy and antitumor immune activation. The hematoxylin and eosin (H&E), Ki‐67, and terminal‐deoxynucleotidyl transferase‐mediated nick end labeling (TUNEL) staining showed pronounced necrosis in the tumor tissues from the mouse group receiving the treatment of CD/CpG@LNPs (+ NIR), along with a remarkable reduction in tumor cell proliferation (Figure 5D; Figure S10, Supporting Information). The data support the hypothesis that CD/CpG@LNPs serve as a potent immunoadjuvant‐enhanced phototherapeutic agent.

Next, experiments were conducted to elucidate the underlying mechanisms of the CD/CpG@LNP‐induced anti‐tumor immune response. First, colorectal tumors, spleens, and lymph nodes were collected and assayed for the degrees of DC maturation and T lymphocyte activation via immunofluorescence staining. It was observed that the CD/CpG@LNP (+ NIR) group exhibited the highest fluorescence intensities in mDCs, CD4^+^ T cells, and CD8^+^ T cells among all mouse groups (Figure S11, Supporting Information). Next, we quantified the levels of pro‐inflammatory cytokines using an enzyme‐linked immunosorbent assay kit to evaluate the involvement of DCs and CTLs in the tumor tissues.^[^ ^48^ ^]^ It was found that the levels of serum TNF‐*α* in the CD/CpG@LNP (+ NIR) group were the highest among all groups (Figure 5E). These encouraging findings demonstrate that the utilization of CD/CpG@LNPs in conjunction with NIR irradiation effectively induced DC maturation, leading to the enhanced recruitment of CTLs and the secretion of TNF‐*α* for combating colorectal tumors.

To further enhance the therapeutic efficacy of CD/CpG@LNPs against CRC, our focus shifted to conjugating them to the surface of LR for in vivo antitumor effects according to the timeline presented in Figure 5F. No significant difference was observed in the mean body weights among all mouse groups during the treatment period, suggesting minimal adverse effects of the therapeutic modalities (Figure S12, Supporting Information). When compared to LR‐CD/CpG@LNPs (without ROS‐sensitive linkers), the NIR‐assisted LR‐S‐CD/CpG@LNPs exerted a remarkably more potent tumor suppressive effect, as reflected by a significant decrease in both numbers (1.9‐fold lower) and sizes of tumors (Figure 5G,H; Figure S13, Supporting Information). Meanwhile, the superior antitumor outcomes of LR‐S‐CD/CpG@LNP (+ NIR) treatment were confirmed by H&E, Ki‐67, and TUNEL staining of the colorectal tumors (Figure 5I; Figure S14, Supporting Information). The primary reason for these antitumor results is the increased accumulation of CD/CpG@LNPs released from LR‐S‐CD/CpG@LNPs into the ROS‐enriched colorectal tumor tissues, rather than being held outside tumor tissues by aerophilic LRs. Afterward, we explored the impact of LRs on immune responses in the mesenteric lymph nodes and spleens. The highest level of mDCs was found in the LR‐CD/CpG@LNP (+ NIR) group (Figure 5J; Figure S15A, Supporting Information), and these mDCs could assist in recruiting more helpers to attack the colorectal tumor cells. Indeed, the LR‐S‐CD/CpG@LNP (+ NIR) group presented 1.3‐fold and 1.6‐fold increases in the aggregates of CD3^+^CD8^+^ T cells compared to the CD/CpG@LNP (+ NIR) and LR‐CD/CpG@LNP (+ NIR) groups, respectively, mirroring the superior activation capability of immune T cells by the treatment of LR‐S‐CD/CpG@LNPs plus NIR irradiation (Figure 5K; Figure S15B, Supporting Information).

### 2.6 Oral LR‐S‐CD/CpG@LNPs for Suppressing Orthotopic CRC and Liver Metastasis

**Figure 6:**
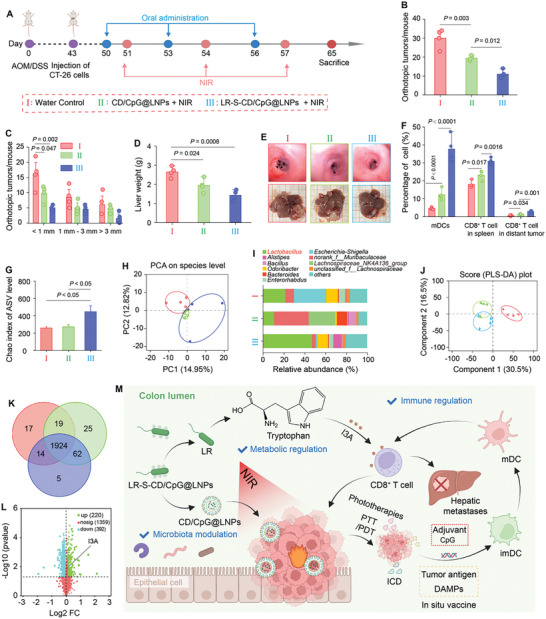
In vivo therapeutic outcomes, intestinal microbiota, and metabolic regulation of various treatment modalities against orthotopic CRC and hepatic metastases. A) Schematic illustration of tumor model establishment and treatment procedures against orthotopic colorectal tumors and hepatic metastases. B) Orthotopic tumor numbers and C) tumor size distributions at the end of treatments. D) Liver weights of all mouse groups at the end of treatments. E) Endoscopic images of colorectal tumors and liver tumor images from various treatment groups. F) Percentages of DCs and CD8^+^ T cells in the tumor‐draining lymph nodes, spleens, and liver tumors. G) Chao indices of microbes at the ASV levels. H) Principle component analysis (PCA) plots of various mouse groups. I) The relative abundance of the fecal bacterial genus. J) PLS‐DA analysis of the water control, CD/CpG@LNP, and LR‐S‐CD/CpG@LNP groups. K) Venn diagram displaying (comparatively) the differentially expressed metabolites. L) Volcanic maps of intestinal metabolites between the water control and LR‐S‐CD/CpG@LNP groups. M) The therapeutic mechanism of LR‐S‐CD/CpG@LNPs against orthotopic colorectal tumors and hepatic metastases while boosting anti‐tumor immunity. Inset (M) was created with BioRender.com. (n = 4 biologically independent experiments; *p < 0.05, **p < 0.01, ***p < 0.001, and ****p < 0.0001 by one‐way ANOVA with Tukey's multiple comparison test).

The liver has been identified as a preferred organ for the metastasis of colorectal tumors, which is attributed to the favorable interaction between colon tumor cells and the specific microenvironment of hepatic tissues.^[^ ^17^, ^49^ ^]^ We wondered whether the LR‐S‐CD/CpG@LNP‐induced photo‐immunotherapeutic treatment could elicit potent antitumor immunity to inhibit metastatic tumors in the liver. To validate this, an orthotopic CRC mouse model with liver metastases was established. Following various treatments in accordance with the protocol outlined in **Figure** **6**A, the physical characteristics of both primary and distant tumors were determined. The body weights of all mouse groups remained similar (Figure S16, Supporting Information). The treatment with LR‐S‐CD/CpG@LNPs (+ NIR) not only suppressed the growth of orthotopic colorectal tumors (Figure 6B,C), but also inhibited the development of distant liver tumors, which could be seen from a significant reduction in liver weights (Figure 6D) and tumor sizes (Figure 6E). The results obtained from H&E, IFN‐*γ*, Ki67, and TUNEL staining collectively reflected the antitumor effect of LR‐S‐CD/CpG@LNPs (+ NIR) on both primary and distant tumors, as characterized by predominant tumor cell necrosis, the abundant presence of IFN‐*γ*, induction of apoptosis, and inhibition of tumor cell proliferation (Figure S17, Supporting Information). Furthermore, this treatment modality resulted in the augmentation of spleen weights, suggesting its capacity to potentiate the antitumor immune responses (Figure S18, Supporting Information). The FCM results depicted in Figures 6F and S19 (Supporting Information) demonstrated that LR‐S‐CD/CpG@LNPs (+ NIR) significantly induced DC maturation in the lymph nodes, as well as activation of CD8^+^ T cells in the spleens and distant tumors when compared to the treatment of CD/CpG@LNPs (+ NIR).

### 2.7 Oral LR‐S‐CD/CpG@LNPs Alter Recipient Gut Microbiota

Accumulating reports have demonstrated that the gut microbial system is associated with CRC development.^[^ ^50^, ^51^ ^]^ Building upon this, we comprehensively assessed the impact of LR‐S‐CD/CpG@LNPs (+ NIR) on the microbial compositions. The Chao (Figure 6G) and Shannon (Figure S20A, Supporting Information) indices of gut microbiota were upregulated after treatment with LR‐S‐CD/CpG@LNPs (+ NIR), indicating their potential to aggrandize bacterial diversity. Moreover, we found that the LR‐S‐CD/CpG@LNP (+ NIR) group revealed a distinct differentiation in the intestinal microbiota from the water control and CD/CpG@LNP (+ NIR) groups, as indicated by principal component analysis (PCA) based on species (Figure 6H). The Venn diagram (Figure S20B, Supporting Information) and genus‐level composition analysis (Figure 6I) proved that the LR‐S‐CD/CpG@LNP (+ NIR) treatment increased the diversity of intestinal microbiota with *Lactobacillus* as the predominant species. At the species level (Figure S20C, Supporting Information), the treatment with LR‐S‐CD/CpG@LNP (+ NIR) substantially enhanced the abundances of beneficial bacteria (e.g., *Lachnospiraceae_UCG‐006* and *Alistipes*), while reducing the proportions of harmful bacteria (e.g., *Enterobaçteriaceae*).

### 2.8 Oral LR‐S‐CD/CpG@LNPs Enhance the Immune Response by Regulating Tryptophan Metabolism to Increase I3A Levels

I3A, a typical immune‐modulatory metabolite, is released by *Lactobacillus* through tryptophan decomposition, which has been reported to activate the immune system by binding to the AHR.^[^ ^13^, ^52^, ^53^ ^]^ Given the upregulated abundance of *Lactobacillus* in the LR‐S‐CD/CpG@LNP (+ NIR) group, we performed nonspecific metabolomics analysis on fecal samples. The discriminant analysis using partial least squares discriminant analysis (PLS‐DA) revealed a separated clustering between the water control group and the LR‐S‐CD/CpG@LNP (+ NIR) group (Figure 6J). The Venn diagram analysis was performed to determine the differential metabolites, showcasing 63 differentially‐produced metabolites between the CD/CpG@LNP (+ NIR) group and the LR‐S‐CD/CpG@LNP (+ NIR) group (Figure 6K). The volcano plots revealed an upregulation of I3A levels following treatment with LR‐S‐CD/CpG@LNPs plus NIR irradiation compared to the water control (Figure 6L). Subsequently, nonspecific metabolomics identified tryptophan metabolism as a pathway with a 2.0‐fold significant enrichment (Figure S21A, Supporting Information). Treatment with LR‐S‐CD/CpG@LNPs (+ NIR) decreased the abundance of tryptophan, revealing that this treatment modality caused its degradation compared with the CD/CpG@LNP (+ NIR) group (Figure S21B, Supporting Information). These collective findings suggest that *Lactobacillus* plays a facilitating role in tryptophan metabolism, producing I3A, thereby assisting the immunotherapeutic responses of orthotopic colorectal tumors and inhibiting tumor metastasis (Figure 6M).

## 3 Conclusion

Immunotherapy has revolutionized the field of cancer treatment by harnessing the immune system to selectively target malignant tumors.^[^ ^54^ ^]^ However, the efficacy of conventional immunotherapy is limited by various challenges, including immune evasion mechanisms, tumor cell heterogeneity, and obstacles in achieving precise drug delivery, resulting in a restricted or absent response in over 80% of patients.^[^ ^55^ ^]^ Alternatively, certain specific bacteria have emerged as victorious agents in tumor immunotherapy by capitalizing on their unique ability to naturally elicit innate immune responses.^[^ ^56^, ^57^ ^]^ Despite its efficacy in enhancing treatment outcomes, intravenous injection of bacteria raises significant safety concerns due to its potential to trigger uncontrolled systemic immune responses, thereby leading to acute inflammation and an elevated risk of thrombosis. In contrast, oral administration emerges as the most convenient route for CRC treatment.^[^ ^58^ ^]^ Nonetheless, its suitability in a complex gastrointestinal environment must be taken into consideration.^[^ ^59^ ^]^

Here, we developed a safe and effective ROS‐responsive bacterium‐based platform armed with CD/immunoadjuvant‐co‐loaded LNPs, enabling the simultaneous application of phototherapy and immunoadjuvant‐ and bacteria‐enhanced immunotherapy. CD/CpG@LNPs were accumulated in the colorectal tumors with the assistance of LR; the responsively released CD/CpG@LNPs penetrated further into the colorectal tumor tissues. With the excellent phototherapeutic effect under NIR irradiation, CD/CpG@LNPs achieved photothermal ablation, amplified oxidative stress, induced ICD burst, and stimulated DC maturation. In vivo results revealed that LR‐S‐CD/CpG@LNP treatment effectively increased the abundance of *Lactobacillus*, upregulating the level of immunomodulatory I3A through the tryptophan metabolic pathway. The remarkable phototherapeutic and immune activation efficacies of LR‐S‐CD/CpG@LNPs (+ NIR) effectively suppressed orthotopic colorectal tumors and metastatic live tumors, demonstrating their promising translational potential in treating metastatic CRC.

## 4 Experimental Section

### Extraction and Purification of MLLs

Mulberry leaves were washed with water and dried in an oven at 37 °C for 24 h. The leaves were pulverized into fragments using a micro mill and sieved through 100 mesh screens. The resulting powder was extracted with cyclohexane solutions at a liquid‐to‐material ratio of 20:1 for 3 h. Organic solvent was collected, followed by the purification and separation of lipids utilizing column chromatography. Finally, the total fat‐soluble contents (MLLs) were obtained by evaporating the organic solvents under reduced pressure at 30 °C.

### Preparation of CD/CpG@LNPs

MLLs (20 mg) and p127 (4 mg) were dissolved in 2 mL of ethanol and dried into a film at 37 °C using a rotary evaporator.^[^ ^38^ ^]^ Subsequently, a round‐bottom flask containing the MLL/p127 film was supplemented with CD (2 mg) and CpG (100 µg) aqueous solution, followed by sonication in a water bath at 100 W for 2 min. Finally, the CD/CpG@LNP suspensions were collected and stored at −20 °C.

### Preparation of LR‐S‐CD/CpG@LNPs

Briefly, 1 mL of CD/CpG@LNP suspension (400 µg mL^−1^) was activated by incubating with 1‐(3‐dimethylaminopropyl)−3‐ethylcarbodiimide (EDC), *N*‐hydroxysulfosuccinimide sodium salt (NHS), and thiodiglycolic acid for 1 h in 3 mL of PBS buffer (pH 5.5) at room temperature.^[^ ^60^, ^61^ ^]^ Bioconjugation was achieved by incubating ≈10^8^ CFU of LR with NHS‐activated S‐CD/CpG@LNPs in PBS for 3 h at 37 °C under gentle agitation, facilitating amide coupling. Next, centrifugation at a force of 1500 g was proceeded to remove the unbound CD/CpG@LNPs, followed by three rinses with PBS. Finally, the samples were resuspended in PBS with a pH of 7.4.

### Cell Viability Assay

The viability of CT‐26 cells was assessed using an MTT assay. CT‐26 cell suspensions (1 × 10^4^ cells/well) were seeded into a 96‐well plate and incubated for 12 h in a humidified incubator (37 °C and 5% CO_2_). After discarding the supernatant, different concentrations of CD@LNP and CD/CpG@LNP suspensions were added. Following a 4‐h incubation period, the wells in the NIR groups were exposed to irradiation (0.5 W cm^−2^ for 3 min), and the cells were cultured for an additional 20 h. The absorbance of all cells at 570 nm was measured using a microplate reader (BioTek, USA).

### In Vitro Detection of ICD

CT‐26 cells were seeded into 24‐well culture plates at a density of 1 × 10^5^/well and incubated at 37 °C for 12 h. Subsequently, cells were treated with CD@LNPs and CD/CpG@LNPs. After a 4‐h incubation, cells underwent irradiation with a 660 nm laser (0.5 W cm^−2^, 3 min) followed by continuous incubation for an additional 20 h. Following this, cells were fixed with formaldehyde (4%, v v^−1^) for 10 min, rinsed thrice with PBS, permeabilized using Triton X‐100 (0.1%, w v^−1^) for 5 min, and blocked using BSA solution (1%, w v^−1^) in PBS for 1 h. Next, cells were incubated overnight at 4 °C with primary antibodies targeting HMGB1 or CRT, rinsed three times using PBS, and treated with the corresponding chromophore‐conjugated secondary antibodies. Finally, 4′,6‐diamidino‐2‐phenylindole (DAPI) staining was performed in the cells for 5 min, followed by image processing utilizing CLSM (Zeiss‐800, Germany).

CT‐26 cells were seeded at a density of 5 × 10^5^ cells per well in 6‐well culture plates and incubated at 37 °C for 12 h. Subsequently, cells were treated with CD@LNPs and CD/CpG@LNPs. After a 4‐h incubation, cells underwent irradiation with a 660 nm laser (0.5 W cm^−2^ for 3 min) followed by continuous incubation for an additional 20 h. Afterward, ATP and caspase‐3 levels were quantified using the corresponding assay kits according to the manufacturer's instructions (Beyotime Institute of Biotechnology, Nanjing, Jiangsu, China).

### Animal Tumor Models

Female BALB/c mice (6–8 weeks) were sourced from Chongqing Laibite Biotechnologies Company (Chongqing, China). All procedures involving the mice followed protocols approved by the Institutional Animal Care and Use Committee of Southwest University (IACUC‐20240520‐02). The mouse models with orthotopic CRC were established as follows: the mice received intraperitoneal injections of azoxymethane (AOM) solution at 10 mg kg^−1^. Seven days after injection, the mice underwent two cycles of dextran sulfate sodium salt (DSS) treatment, each consisting of a 2.5% (w v^−1^) DSS solution for 7 days, followed by a 14‐day recovery period with regular water. The establishment of a double tumor mouse model with orthotopic CRC and distant liver metastasis was performed by inoculating suspended CT‐26 cells (25 µL, 1 × 10^7^ cells mL^−1^) into the left lobe of the liver of mice bearing in situ CRC.

### In Vivo Bio‐Distribution of LR‐S‐CD/CpG@LNPs

LR‐S‐CD/CpG@LNPs were orally administered to mice bearing orthotopic CRC at a 3 mg kg^−1^ CD concentration. Subsequently, mice were euthanized at 6, 12, 24, 48, and 72 h after administration to collect the GITs as well as other principal organs, including heart, liver, spleen, lung, and kidney. The organs were imaged using a live imaging system (FOBI; NeoScience, Republic of Korea). Furthermore, colorectal tissues were embedded in optimal cutting temperature (OCT) compound followed by sectioning at a thickness of 5 µm. These sections were stained with DAPI for 5 min for image processing using CLSM (Zeiss‐800, Germany).

### In Vivo Therapeutic Outcomes of CD/CpG@LNPs against Orthotropic CRC

To assess the in vivo anti‐tumor efficacy of CD/CpG@LNPs, orthotopic tumor mouse model was established. Mice were randomly divided into five groups: water control, CD@LNPs, CD@LNPs (+ NIR), CD/CpG@LNPs, and CD/CpG@LNPs (+ NIR). After oral administration of various NPs (CD: 3 mg kg^−1^; CpG: 16 µg; 200 µL) for 24 h, mice involving NIR groups were subjected to NIR irradiation for 3 min at a power density of 1 W cm^−2^. On day 16, mice were sacrificed. Colorectal tumors were counted using a dissecting microscope (Olympus, SZ40) and subjected to H&E and immunofluorescence staining for Ki67 and TUNEL. Additionally, immunofluorescence staining of colorectal tumors and spleens was performed for either CD4^+^ or CD8^+^ T cell markers.

### In Vivo Therapeutic Outcomes of CD/CpG@LNPs against Orthotropic CRC and Liver Metastasis

After establishing the orthotopic CRC mouse model, CT‐26 cells (25 µL, 1 × 10^7^ cells/mL) were injected into the left lobe of the liver in each mouse. Three days post‐injection, mice were randomly divided into three groups: the water control group, the CD/CpG@LNP (+ NIR) group, and the LR‐S‐CD/CpG@LNP (+ NIR) group. Twenty‐four hours after oral administration of H_2_O, CD/CpG@LNPs, and LR‐S‐CD/CpG@LNPs (CD, 3 mg kg^−1^; CpG, 16 µg; 200 µL), mice in the NIR‐involved groups were irradiated for 3 min with an NIR laser (660 nm, 1 W cm^−2^). At the end of the treatment, mice were sacrificed. Colonic tumors were counted and measured using a dissecting microscope (Olympus, SZ40). Distant liver tumor tissues were excised, weighed, and photographed. The colon and liver tissues underwent H&E, Ki67, and TUNEL staining. To analyze DC maturation, tumor lymph nodes were enzymatically digested to obtain single‐cell suspensions, which were stained with antibodies against CD11c, CD80, and CD86. To analyze T cell activation, spleens and liver tumors were collected and enzymatically digested to obtain single‐cell suspensions in PBS. These cell suspensions were stained with antibodies against CD3, CD4, and CD8 for detection by FCM (ACEA NovoCyte, USA).

### Statistical Analysis

All values are expressed as mean ± standard error of the mean (s.e.m.). Statistical analysis was conducted using Student's *t*‐test or one‐way analysis of variance, unless otherwise noted. Statistical significance was represented by *p < 0.05, **p < 0.01, ***p < 0.001, and ****p < 0.0001.

## Conflict of Interest

The authors declare no conflict of interest.

## Author Contributions

H.X. and Y.W. contributed equally to this work. R.R., S.K., M.Z., X.S., and B.X. designed experiments, supervised the project, and wrote the manuscript draft. H.X., Y.W., G.L., Z.Z., M.A.S., and M.Z. performed the experiments. H.X., Y.W., G.L., Z.Z., X.S., M.Z., and B.X. co‐wrote the manuscript. Z.Z., X.S., R.R., S.K., and B.X. edited and revised the manuscript. All authors approved the final version of the manuscript.

## Supporting information



Supporting Information

## Data Availability

All data needed to evaluate the conclusions in the paper are present in the paper and/or the Supplementary Materials. Additional data related to this paper may be requested from the authors.

## References

[1] X. Huang , J. Pan , F. Xu , B. Shao , Y. Wang , X. Guo , S. Zhou , Adv. Sci. 2021, 8, 2003572. 10.1002/advs.202003572 PMC8025040 33854892

[2] Y. Guo , Y. Chen , X. Liu , J. J. Min , W. Tan , J. H. Zheng , Cancer Lett. 2020, 469, 102. 31666180 10.1016/j.canlet.2019.10.033

[3] W. Chen , Y. Wang , M. Qin , X. Zhang , Z. Zhang , X. Sun , Z. Gu , ACS Nano 2018, 12, 5995. 29786420 10.1021/acsnano.8b02235

[4] C. R. Gurbatri , I. Lia , R. Vincent , C. Coker , S. Castro , P. M. Treuting , T. E. Hinchliffe , N. Arpaia , T. Danino , Sci. Transl. Med. 2020, 12, eaax0876. 32051224 10.1126/scitranslmed.aax0876 PMC7685004

[5] D. Chandra , A. Jahangir , W. Quispe‐Tintaya , M. H. Einstein , C. Gravekamp , Br. J. Cancer 2013, 108, 2281. 23640395 10.1038/bjc.2013.206 PMC3681012

[6] B. Wei , J. Pan , R. Yuan , B. Shao , Y. Wang , X. Guo , S. Zhou , Nano Lett. 2021, 21, 4231. 33998789 10.1021/acs.nanolett.1c00209

[7] W. Cheng , L. He , W. Ren , T. Yue , X. Xie , J. Sun , X. Chen , Z. Wu , F. Li , J.‐G. Piao , NTM 2023, 2, 100008.

[8] M. R. Park , M. Shin , D. Mun , S. Y. Jeong , D. Y. Jeong , M. Song , G. Ko , T. Unno , Y. Kim , S. Oh , Sci. Rep. 2020, 10, 21701. 33303803 10.1038/s41598-020-77587-w PMC7729874

[9] H. Y. Liu , F. Gu , C. Zhu , L. Yuan , C. Zhu , M. Zhu , J. Yao , P. Hu , Y. Zhang , J. Dicksved , W. Bao , D. Cai , Front. Immunol. 2022, 13, 865982. 35320932 10.3389/fimmu.2022.865982 PMC8934773

[10] J. M. Wells , Microb. Cell Fact. 2011, 10, S17. 21995674 10.1186/1475-2859-10-S1-S17 PMC3231924

[11] Y. Gong , Z. Liu , P. Zhou , J. Li , Y.‐B. Miao , NTM 2023, 2, 100020.

[12] H. M. Roager , T. R. Licht , Nat. Commun. 2018, 9, 3294. 30120222 10.1038/s41467-018-05470-4 PMC6098093

[13] M. J. Bender , A. C. McPherson , C. M. Phelps , S. P. Pandey , C. R. Laughlin , J. H. Shapira , L. M. Sanchez , M. Rana , T. G. Richie , T. S. Mims , A. M. Gocher‐Demske , L. Cervantes‐Barragan , S. J. Mullett , S. L. Gelhaus , T. C. Bruno , N. Cannon , J. A. McCulloch , D. A. A. Vignali , R. Hinterleitner , A. V. Joglekar , J. F. Pierre , S. T. M. Lee , D. Davar , H. M. Zarour , M. Meisel , Cell 2023, 186, 1846. 37028428 10.1016/j.cell.2023.03.011 PMC10148916

[14] Z. Luo , A. Chen , A. Xie , X. Liu , S. Jiang , R. Yu , Front. Immunol. 2023, 14, 1228754. 37638038 10.3389/fimmu.2023.1228754 PMC10450031

[15] L. B. Ivashkiv , Nat. Rev. Immunol. 2018, 18, 545. 29921905 10.1038/s41577-018-0029-z PMC6340644

[16] A. S. Adebayo , K. Agbaje , S. K. Adesina , O. Olajubutu , Pharmaceutics 2023, 15, 2620. 38004598 10.3390/pharmaceutics15112620 PMC10674471

[17] H. Zhou , Z. Liu , Y. Wang , X. Wen , E. H. Amador , L. Yuan , X. Ran , L. Xiong , Y. Ran , W. Chen , Y. Wen , Signal Transduction Targeted Ther. 2022, 7, 70. 10.1038/s41392-022-00922-2 PMC8897452 35246503

[18] Y. H. Xie , Y. X. Chen , J. Y. Fang , Signal Transduction Targeted Ther. 2020, 5, 22. 10.1038/s41392-020-0116-z PMC7082344 32296018

[19] Y. Lu , Y. Gao , H. Yang , Y. Hu , X. Li , Mil. Med. Res. 2022, 9, 69. 36503490 10.1186/s40779-022-00433-9 PMC9743634

[20] K. Ganesh , Z. K. Stadler , A. Cercek , R. B. Mendelsohn , J. Shia , N. H. Segal , L. A. Diaz Jr. , Nat. Rev. Gastroenterol. Hepatol. 2019, 16, 361. 30886395 10.1038/s41575-019-0126-x PMC7295073

[21] P. Agarwal , D. T. Le , P. M. Boland , Adv. Cancer Res. 2021, 151, 137. 34148613 10.1016/bs.acr.2021.03.002

[22] C. Pleguezuelos‐Manzano , J. Puschhof , A. Rosendahl Huber , A. van Hoeck , H. M. Wood , J. Nomburg , C. Gurjao , F. Manders , G. Dalmasso , P. B. Stege , F. L. Paganelli , M. H. Geurts , J. Beumer , T. Mizutani , Y. Miao , R. van der Linden , S. van der Elst , K. C. Garcia , J. Top , R. J. L. Willems , M. Giannakis , R. Bonnet , P. Quirke , M. Meyerson , E. Cuppen , R. van Boxtel , H. Clevers , Nature 2020, 580, 269.32106218

[23] C. M. Dejea , P. Fathi , J. M. Craig , A. Boleij , R. Taddese , A. L. Geis , X. Wu , C. E. DeStefano Shields , E. M. Hechenbleikner , D. L. Huso , R. A. Anders , F. M. Giardiello , E. C. Wick , H. Wang , S. Wu , D. M. Pardoll , F. Housseau , C. L. Sears , Science 2018, 359, 592. 29420293 10.1126/science.aah3648 PMC5881113

[24] R. Qu , Y. Zhang , Y. Ma , X. Zhou , L. Sun , C. Jiang , Z. Zhang , W. Fu , Adv. Sci. 2023, 10, e2205563. 10.1002/advs.202205563 PMC10427379 37263983

[25] H. Simsek , E. Klotzsch , BioEssays 2022, 44, e2100285. 35393714 10.1002/bies.202100285

[26] Y. Jin , Y. Huang , H. Ren , H. Huang , C. Lai , W. Wang , Z. Tong , H. Zhang , W. Wu , C. Liu , X. Bao , W. Fang , H. Li , P. Zhao , X. Dai , Biomaterials 2024, 305, 122463. 38232643 10.1016/j.biomaterials.2023.122463

[27] M. Overchuk , R. A. Weersink , B. C. Wilson , G. Zheng , ACS Nano 2023, 17, 7979. 37129253 10.1021/acsnano.3c00891 PMC10173698

[28] J. Lou , M. Aragaki , N. Bernards , T. Chee , A. Gregor , Y. Hiraishi , T. Ishiwata , C. Leung , L. Ding , S. Kitazawa , T. Koga , Y. Sata , H. Ogawa , J. Chen , T. Kato , K. Yasufuku , G. Zheng , Biomaterials 2023, 292, 121918. 36442438 10.1016/j.biomaterials.2022.121918

[29] Z. Shi , H. Bai , J. Wu , X. Miao , J. Gao , X. Xu , Y. Liu , J. Jiang , J. Yang , J. Zhang , T. Shao , B. Peng , H. Ma , D. Zhu , G. Chen , W. Hu , L. Li , W. Huang , Research 2023, 6, 0169. 37342631 10.34133/research.0169 PMC10278946

[30] J. Zhu , R. Chang , B. Wei , Y. Fu , X. Chen , H. Liu , W. Zhou , Research 2022, 2022, 9816272. 36157510 10.34133/2022/9816272 PMC9484834

[31] J. Galon , D. Bruni , Nat. Rev. Drug Discovery 2019, 18, 197. 30610226 10.1038/s41573-018-0007-y

[32] L. Wang , H. Geng , Y. Liu , L. Liu , Y. Chen , F. Wu , Z. Liu , S. Ling , Y. Wang , L. Zhou , MedComm 2023, 4, e343. 37638340 10.1002/mco2.343 PMC10458686

[33] C. Feng , P. Tan , G. Nie , M. Zhu , Exploration 2023, 3, 20210263. 37933383 10.1002/EXP.20210263 PMC10624393

[34] J. Ouyang , Z. Zhang , B. Deng , J. Liu , L. Wang , H. Liu , S. Koo , S. Chen , Y. Li , A. V. Yaremenko , X. Huang , W. Chen , Y. Lee , W. Tao , Mater. Today 2023, 62, 296.

[35] M. P. Spindler , S. Siu , I. Mogno , Z. Li , C. Yang , S. Mehandru , G. J. Britton , J. J. Faith , Cell Host Microbe 2022, 30, 1481 . 36099923 10.1016/j.chom.2022.08.009 PMC9588646

[36] C. Zhang , W. Gong , Z. Li , D. Gao , Y. Gao , Food Sci. Hum. Wellness 2019, 8, 102.

[37] C. Luo , J. Sun , D. Liu , B. Sun , L. Miao , S. Musetti , J. Li , X. Han , Y. Du , L. Li , L. Huang , Z. He , Nano Lett. 2016, 16, 5401. 27490088 10.1021/acs.nanolett.6b01632 PMC5541379

[38] B. Li , M. Zu , A. Jiang , Y. Cao , J. Wu , M. A. Shahbazi , X. Shi , R. L. Reis , S. C. Kundu , B. Xiao , Biomaterials 2024, 307, 122530. 38493672 10.1016/j.biomaterials.2024.122530

[39] Y. Wang , Y. Chen , X. Zhang , Y. Lu , H. Chen , J. Funct. Foods 2020, 75, 104248.

[40] W. Wang , M. Green , J. E. Choi , M. Gijón , P. D. Kennedy , J. K. Johnson , P. Liao , X. Lang , I. Kryczek , A. Sell , H. Xia , J. Zhou , G. Li , J. Li , W. Li , S. Wei , L. Vatan , H. Zhang , W. Szeliga , W. Gu , R. Liu , T. S. Lawrence , C. Lamb , Y. Tanno , M. Cieslik , E. Stone , G. Georgiou , T. A. Chan , A. Chinnaiyan , W. Zou , Nature 2019, 569, 270. 31043744 10.1038/s41586-019-1170-y PMC6533917

[41] L. Xiao , M. Xian , C. Zhang , Q. Guo , Q. Yi , Front. Immunol. 2023, 14, 1322746. 38259464 10.3389/fimmu.2023.1322746 PMC10800824

[42] R. Alzeibak , T. A. Mishchenko , N. Y. Shilyagina , I. V. Balalaeva , M. V. Vedunova , D. V. Krysko , J Immunother Cancer 2021, 9, e001926corr1. 33431631 10.1136/jitc-2020-001926 PMC7802670

[43] C. Volpi , F. Fallarino , M. T. Pallotta , R. Bianchi , C. Vacca , M. L. Belladonna , C. Orabona , A. De Luca , L. Boon , L. Romani , U. Grohmann , P. Puccetti , Nat. Commun. 2013, 4, 1852. 23673637 10.1038/ncomms2874

[44] Q. Ni , F. Zhang , Y. Liu , Z. Wang , G. Yu , B. Liang , G. Niu , T. Su , G. Zhu , G. Lu , L. Zhang , X. Chen , Sci. Adv. 2020, 6, eaaw6071. 32206706 10.1126/sciadv.aaw6071 PMC7080439

[45] D. M. Klinman , Nat. Rev. Immunol. 2004, 4, 249. 15057783 10.1038/nri1329

[46] W. Pei , L. Cai , X. Gong , L. Zhang , J. Zhang , P. Zhu , H. Jiang , C. Wang , S. Wang , J. Chen , Mater. Today Bio 2022, 15, 100272. 10.1016/j.mtbio.2022.100272 PMC9123267 35607417

[47] B. Parang , C. W. Barrett , C. S. Williams , Methods Mol. Biol. 2016, 1422, 297. 27246042 10.1007/978-1-4939-3603-8_26 PMC5035391

[48] Z. Li , X. Lai , S. Fu , L. Ren , H. Cai , H. Zhang , Z. Gu , X. Ma , K. Luo , Adv. Sci. 2022, 9, e2201734. 10.1002/advs.202201734 PMC9353475 35652198

[49] D. I. Tsilimigras , P. Brodt , P.‐A. Clavien , R. J. Muschel , M. I. D'Angelica , I. Endo , R. W. Parks , M. Doyle , E. de Santibañes , T. M. Pawlik , Nat. Rev. Dis. Primers 2021, 7, 27. 33859205 10.1038/s41572-021-00261-6

[50] W. Cui , M. Guo , D. Liu , P. Xiao , C. Yang , H. Huang , C. Liang , Y. Yang , X. Fu , Y. Zhang , J. Liu , S. Shi , J. Cong , Z. Han , Y. Xu , L. Du , C. Yin , Y. Zhang , J. Sun , W. Gu , R. Chai , S. Zhu , B. Chu , Nat. Cell Biol. 2024, 26, 124. 38168770 10.1038/s41556-023-01314-6

[51] W. S. Garrett , Science 2019, 364, 1133. 31221845 10.1126/science.aaw2367

[52] T. Zelante , R. G. Iannitti , C. Cunha , A. De Luca , G. Giovannini , G. Pieraccini , R. Zecchi , C. D'Angelo , C. Massi‐Benedetti , F. Fallarino , A. Carvalho , P. Puccetti , L. Romani , Immunity 2013, 39, 372. 23973224 10.1016/j.immuni.2013.08.003

[53] L. Cervantes‐Barragan , J. N. Chai , M. D. Tianero , B. Di Luccia , P. P. Ahern , J. Merriman , V. S. Cortez , M. G. Caparon , M. S. Donia , S. Gilfillan , M. Cella , J. I. Gordon , C. S. Hsieh , M. Colonna , Science 2017, 357, 806. 28775213 10.1126/science.aah5825 PMC5687812

[54] L. Galluzzi , T. A. Chan , G. Kroemer , J. D. Wolchok , A. López‐Soto , Sci. Transl. Med. 2018, 10, eaat7807. 30232229 10.1126/scitranslmed.aat7807

[55] L. Kraehenbuehl , C. H. Weng , S. Eghbali , J. D. Wolchok , T. Merghoub , Nat. Rev. Clin. Oncol. 2022, 19, 37. 34580473 10.1038/s41571-021-00552-7

[56] M. T. Niu , Q. W. Chen , Z. Chen , X. Liu , Q. X. Huang , J. L. Liang , Z. Zhong , H. Cheng , X. Z. Zhang , Nano Lett. 2024, 24, 130. 38150297 10.1021/acs.nanolett.3c03191

[57] P. Feng , Z. Cao , X. Wang , J. Li , J. Liu , Adv. Mater. 2020, 32, 2002406. 10.1002/adma.202002406 32686247

[58] J. Xu , Y. Zhang , J. Xu , M. Wang , G. Liu , J. Wang , X. Zhao , Y. Qi , J. Shi , K. Cheng , Y. Li , S. Qi , G. Nie , Biomaterials 2019, 216, 119. 10.1016/j.biomaterials.2019.119247 31200145

[59] M. Zu , Y. Ma , J. Zhang , J. Sun , M. A. Shahbazi , G. Pan , R. L. Reis , S. C. Kundu , J. Liu , B. Xiao , ACS Nano 2024, 18, 3651. 38241481 10.1021/acsnano.3c11436

[60] B. Wang , X. Zhuang , Z. B. Deng , H. Jiang , J. Mu , Q. Wang , X. Xiang , H. Guo , L. Zhang , G. Dryden , J. Yan , D. Miller , H. G. Zhang , Mol. Ther. 2014, 22, 522. 23939022 10.1038/mt.2013.190 PMC3944329

[61] M. B. Akolpoglu , Y. Alapan , N. O. Dogan , S. F. Baltaci , O. Yasa , G. Aybar Tural , M. Sitti , Sci. Adv. 2022, 8, eabo6163. 35857516 10.1126/sciadv.abo6163 PMC9286503

