# Supplementary material for: Nano‐Armed Limosilactobacillus reuteri for Enhanced Photo‐Immunotherapy and Microbiota Tryptophan Metabolism against Colorectal Cancer
Source: Adv Sci (Weinh). 2024 Dec 30;12(7):2410011. doi: 10.1002/advs.202410011 (PMC11831460; doi:10.1002/advs.202410011)
Supplement: Supplementary file 1 — Supporting Information [file ADVS-12-2410011-s001.docx]

**Supplementary Information**

**Nano-Armed *Limosilactobacillus reuteri* for Enhanced Photo-Immunotherapy and Microbiota Tryptophan Metabolism against Colorectal Cancer**

*Haiting Xu, Yajun Wang, Ga Liu, Zhenhua Zhu, Mohammad-Ali Shahbazi, Rui L. Reis, Subhas C. Kundu, Xiaoxiao Shi*, Menghang Zu*, and Bo Xiao**

H Xu, G Liu, Prof. X Shi, and M Zu

State Key Laboratory of Resource Insects

College of Sericulture, Textile, and Biomass Sciences

Southwest University

Chongqing 400715, China

E-mail: xxshi7@swu.edu.cn; menghangzu@email.swu.edu.cn

Y. Wang and Prof. B. Xiao

Department of Pharmacy

Personalized Drug Therapy Key Laboratory of Sichuan Province

Sichuan Academy of Medical Sciences & Sichuan Provincial People’s Hospital

School of Medicine

University of Electronic Science and Technology

Chengdu 610054, China

E-mail: bxiao@uestc.edu.cn

Prof. Z. Zhu

Department of Gastroenterology

The First Affiliated Hospital of Nanchang University

Nanchang 330006, China

M. Shahbazi

Department of Biomedical Engineering

University Medical Center Groningen

University of Groningen

Antonius Deusinglaan 1, 9713 AV Groningen, Netherlands

W.J. Kolff Institute for Biomedical Engineering and Materials Science

University of Groningen

Antonius Deusinglaan 1, 9713 AV Groningen, Netherlands

Prof. R. Reis and Prof. S. Kundu

3Bs Research Group

I3Bs — Research Institute on Biomaterials, Biodegradables and Biomimetics

University of Minho

Headquarters of the European Institute of Excellence on Tissue Engineering and Regenerative Medicine

AvePark, Barco, Guimarães 4805-017, Portugal

Prof. R. Reis and Prof. S. Kundu

ICVS/3B’s-PT Government Associate Laboratory

Braga, Guimarães 4800-058, Portugal

**Experimental Section**

*Materials*

Chemical reagents, including EDC, NHS, ethanol, and dichloromethane were purchased from Aladdin Chemical Co. Ltd (Shanghai, China). Mulberry leaves were provided by the State Key Laboratory of Silkworm Genome Biology, Southwest University (Chongqing, China). LR was obtained from BeNa Chuanglian Biotechnology Co., Ltd (Beijing, China). CpG was customized from Shanghai Sangon Biological Engineering Technology Services Co., Ltd. The TNF-*α* ELISA Kit, MTT, and live/dead staining kit were obtained from Beijing Solarbio Science & Technology Co., Ltd (Beijing, China). The ROS assay kit, LPO assay kit, GSH assay kit, ATP determination kit, caspase-3 assay kit, and DAPI were provided by Beyotime Institute of Biotechnology (Nanjing Jiangsu, China). The rabbit monoclonal antibodies against CRT and HMGB1 were from Cell Signaling Technology (Danvers, MA, USA). Other antibodies against cell surface markers for FCM (ACEA NovoCyte™, USA) assay were received from eBioscience (California, USA).

*Synthesis of CDs*

CDs were synthesized using mulberry leaves as the biomass carbon source, following the established methodology described in previous studies.^[1]^ Initially, mulberry leaves were finely powdered using a micro-mill. The obtained powders were suspended in ethanol and subjected to stirring for 4 h. After being passed through a filter paper with a pore size of 0.22 μm, the collected filtrate underwent a reaction within an autoclave maintained at a temperature of 150 ℃ for 4 h. The solution was purified by filtration through a polyethersulfone membrane with a pore size of 0.22 μm and concentrated using rotary evaporation (REV212M-B, Yamato Scientific Co., Ltd., Japan). The concentrated product was partitioned between dichloromethane and water in an equal volume ratio. Upon settling, two distinct layers were formed, with the lower layer exhibiting a pronounced green coloration indicative of the presence of the resultant CDs.

*Physicochemical Characterization of* *LR-S-**CD/CpG@LNPs*

The morphologies of CD/CpG@LNPs, LR, and LR-S-CD/CpG@LNPs were examined using TEM (H7700s, Hitachi, Japan). The samples were deposited onto a carbon-coated copper grid and rinsed with deionized water three times. The particle sizes, size distribution profiles, and zeta potentials of CD/CpG@LNPs, LR, and LR-S-CD/CpG@LNPs were determined using DLS with a NanoBrook 90 Plus Zeta instrument (Brookhaven Instrument Corp., New York, USA). The attachment of CD/CpG@LNPs onto the bacterial surface was confirmed by analyzing LR-S-CD/CpG@LNPs using CLSM (Zeiss-800, Germany) and FCM (ACEA NovoCyte™, USA).

*Assessment of Bacterial Viability*

LRs with and without the attachment of CD/CpG@LNPs were suspended in MRS medium (LR concentration = 4 × 10^7^ CFU mL^-1^) and incubated at 37 ℃ with gentle agitation. The optical density (OD) values of the cultures were measured at 600 nm using a microplate reader (BioTek, USA) at predetermined intervals.

*In Vitro Gastrointestinal Stability*

LRs with and without the attachment of CD/CpG@LNPs were suspended in 5 mL of medium supplemented with simulated gastric fluid (pH 2.0), simulated intestinal fluid (pH 6.8), and simulated colonic fluid (pH 6.0). Following incubation at 37 ℃ with gentle agitation for predetermined time intervals, 100 μL of each sample was transferred to a 96-well plate. The OD values of the culture media were measured at a wavelength of 600 nm to assess the viability of LR in different simulated gastrointestinal fluid.

*Live and Dead Cell Staining*

Cell viability was assessed using Calcein AM and PI (propidium iodide) co-staining, following the manufacturer's instructions. Briefly, cells were cultured in 12-well plates at a density of 1 × 10^5^ cells per well. After a 12-h incubation, cells were exposed to the medium containing various samples and cultured for 4 h. This was followed by NIR irradiation at an intensity of 0.5 W cm^-2^ for 3 min, after which the cells were cultured for an additional 20 h. Cells were washed and stained with Calcein AM (for live cell identification) and sodium propidium iodide (for dead cell identification). The stained cells were observed using a CLSM (Zeiss-800, Germany).

*Intracellular ROS Detection*

For the detection of intracellular ROS, DCFH-DA was utilized to assess its levels in CT-26 cells following treatment with CD@LNPs, CD/CpG@LNPs, CD@LNPs (+ NIR), and CD/CpG@LNPs (+ NIR) for 24 h. NIR irradiation was applied for 3 min under the condition of 0.5 W cm^-2^. The stained cells were observed using a CLSM (Zeiss-800, Germany).

*Intracellular GSH* *Detection*

CT-26 cells were seeded into 12-well culture plates at a density of 1 × 10^5^ cells/well and incubated at 37 ℃ for 12 h. Subsequently, the cells were treated with CD@LNPs and CD/CpG@LNPs. After a 4-h incubation, cells underwent irradiation with a 660 nm laser (0.5 W cm^-2^ for 3 min), followed by an additional incubation period of 20 h. The medium was removed, and the cells were washed three times with PBS. 100 μL of lysis buffer containing Triton-X-100 (0.1%, w v^-1^) was used to lyse the cells. The intracellular GSH contents were quantified using a GSH assay kit according to the manufacturer's instructions.

*Intracellular LPO Detection*

CT-26 cells were seeded into 12-well culture plates at a density of 1 × 10^5^ cells/well and incubated at 37 ℃ for 12 h. Subsequently, cells were treated with CD@LNPs and CD/CpG@LNPs. After a 4-h incubation, cells underwent 660 nm laser irradiation (0.5 W cm^-2^, 3 min) followed by an additional incubation period of 20 h. Cells were washed with PBS, and the LPO content was determined using an LPO assay kit according to the manufacturer's instructions.

*In Vitro Induction of DC Maturation*

CT-26 cells were seeded into 12-well culture plates at 1 × 10^5^ cells/well and incubated at 37 ^o^C for 12 h. Subsequently, cells were treated with CD@LNPs and CD/CpG@LNPs. After a 4-h incubation period, cells were subjected to 660 nm NIR irradiation (0.5 W cm^-2^, 3 min) and continuously incubated for 20 h. Then, 1 × 10^5^ immature DCs were co-cultured with the pretreated CT-26 cell culture medium for 24 h. After incubation with anti-CD80-PE and anti-CD86-APC antibodies, DC maturation was examined by FCM (ACEA NovoCyte™, USA).

*In Vivo Antitumor Evaluation of LR-S-CD/CpG@LNPs against Orthotropic CRC*

Mice bearing orthotropic CRC were randomly allocated into five groups: Water control, LR, CD/CpG@LNPs (+ NIR), LR-CD/CpG@LNPs (+ NIR), and LR-S-CD/CpG@LNPs (+ NIR). After oral administration of various therapeutic modalities (CD: 3 mg kg^-1^; CpG: 16 μg; 200 μL) for a duration of 24 h, mice in the NIR-involved groups were subjected to NIR irradiation for 3 min at a power density of 1 W cm^-2^. On day 16, the mice were euthanized. Colonic tumors were counted using a dissecting microscope and subjected to H&E, Ki67, and TUNEL staining. The celiac lymph nodes were collected and digested to obtain single-cell suspensions and determine DC maturation profiles. Specially, these cell suspensions were stained with FITC-labeled anti-CD11c, APC-labeled anti-CD80, and PE-labeled anti-CD86 antibodies, before being analyzed by FCM (ACEA NovoCyte™, USA). To investigate the variations of cytotoxic T cells, the spleens were harvested and digested to obtain single-cell suspensions in the PBS solution. Subsequently, cells were analyzed using FCM (ACEA NovoCyte™, USA) after being stained with FITC-labeled anti-CD3, PE-labeled anti-CD4, and PerCP-labeled anti-CD8 antibodies.


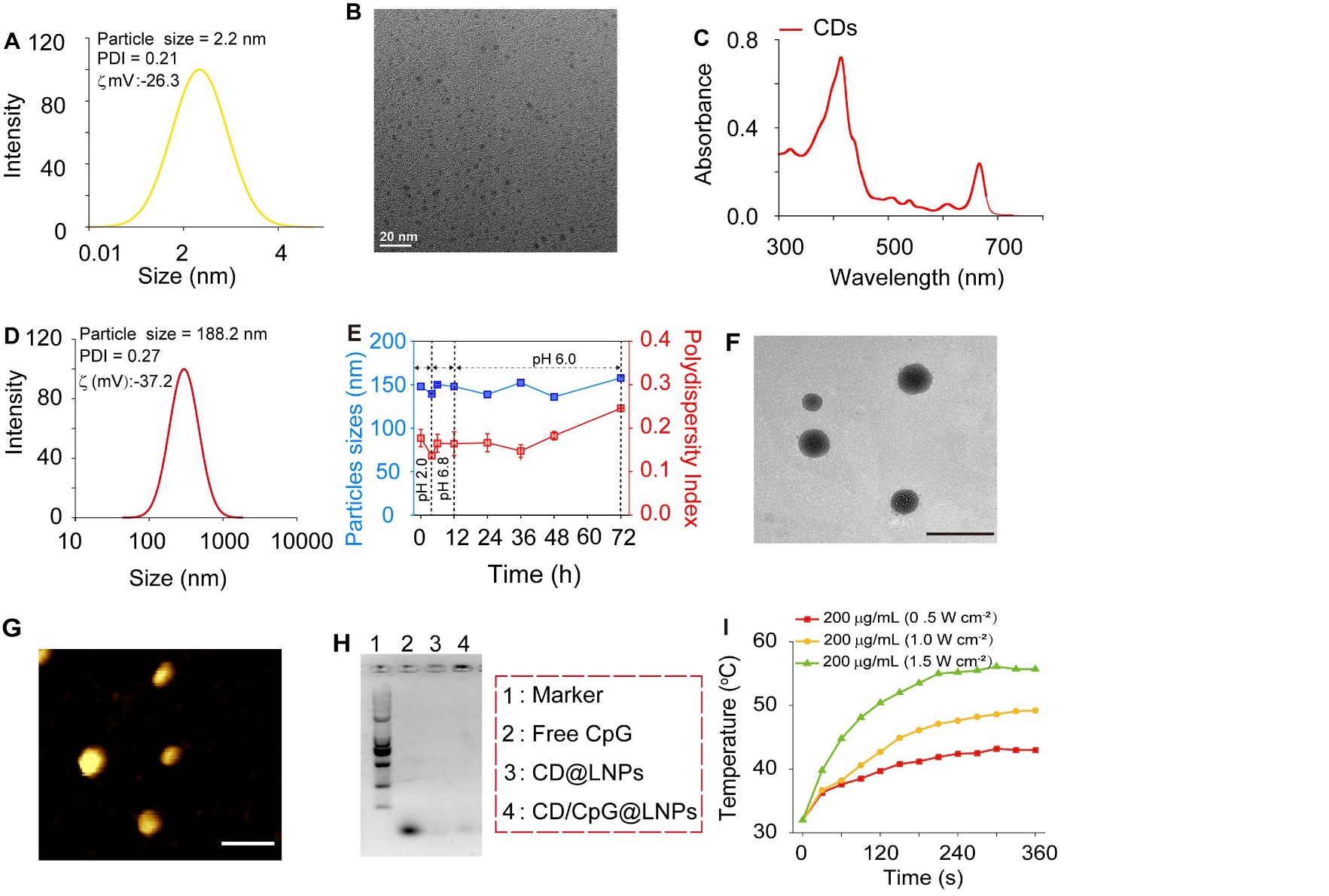


**Figure S1.** (A) Size distribution of CDs measured by DLS. (B) Representative TEM image of CDs. Scale bar: 20 nm. (C) Ultraviolet absorption spectrum of CDs. (D) Size distribution of CD/CpG@LNPs in water. (E) Particle size changes of CD/CpG@LNPs in the simulated gastrointestinal tract fluid at 37 ^o^C (n = 3 independent experiments; Data are expressed as means ± s.e.m). (F) TEM images of CD/CpG@LNPs. Scale bar: 500 nm. (G) AFM images of CD/CpG@LNPs. Scale bar: 500 nm. (H) Agarose gel retardation assay. (I) Laser power-dependent temperature elevation of CD/CpG@LNPs solution (200 μg mL^-1^).


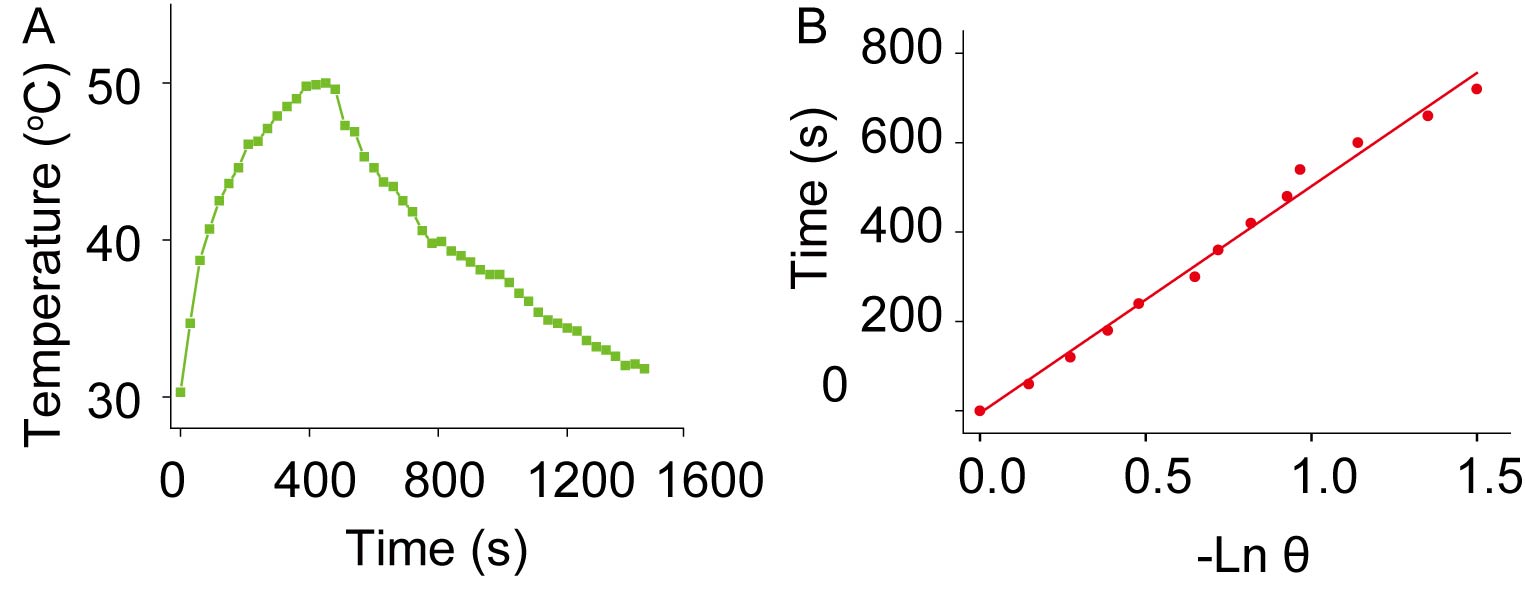


**Figure S2.** (A) Photothermal effect of CD/CpG@LNPs suspensions exposed to 660 nm NIR irradiation (1.0 W cm^-2^) for 10 min. (B) Linear time data from the cooling period versus −ln θ.


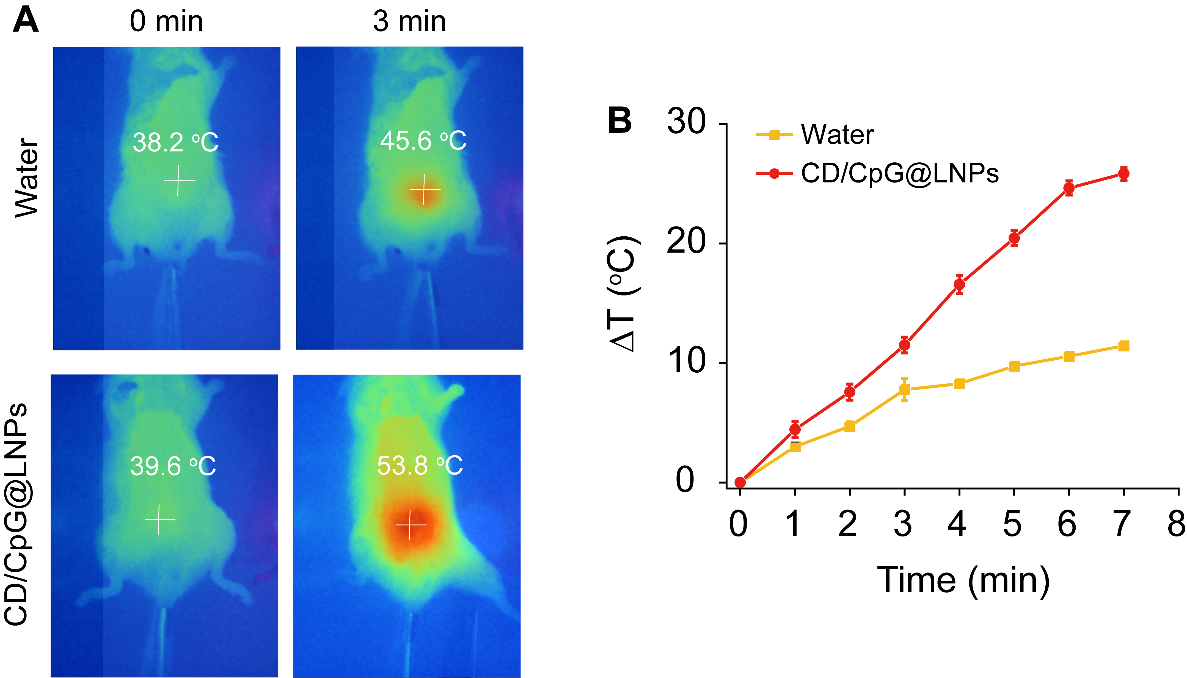


**Figure S3.** (A) Representative photothermal images of mice treated with water and CD/CpG@LNPs (3 mg kg^-1^) with 660 nm NIR irradiation at 1.0 W·cm^-2^ for 3 min. (B) Temperature curves of CRC mice treated with H_2_O and CD/CpG@LNPs (3 mg kg^-1^) with 660 nm NIR irradiation at 1.0 W·cm^-2^ for 7 min (n = 3 independent experiments; Data are expressed as means ± s.e.m).


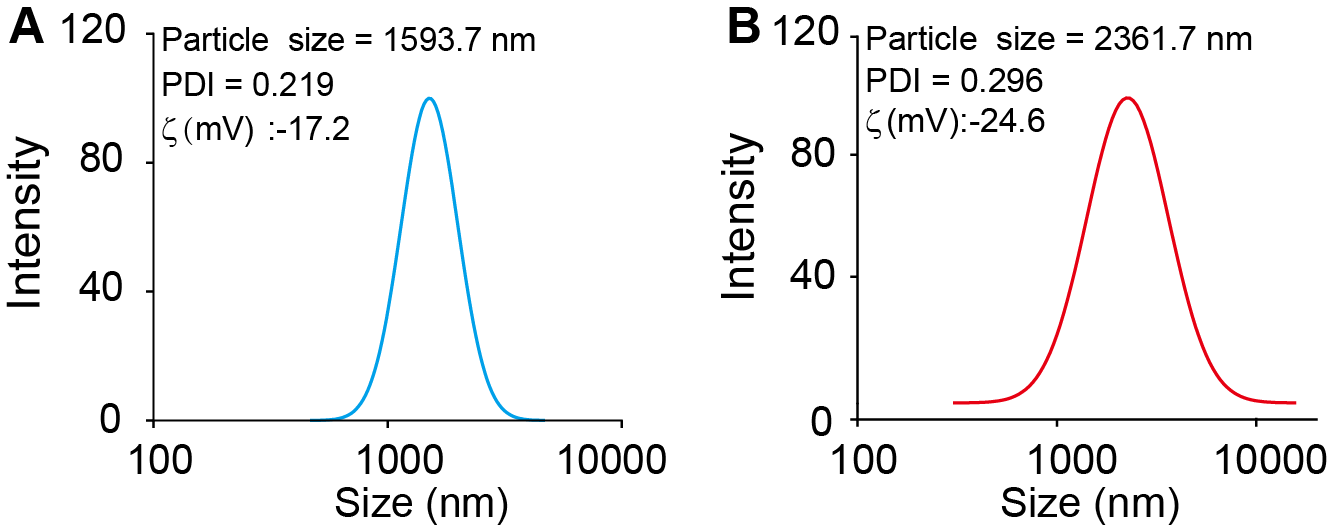


**Figure S4.** Size distribution profiles and zeta potentials of (A) LR and (B) LR-S-CD/CpG@LNPs measured by DLS.


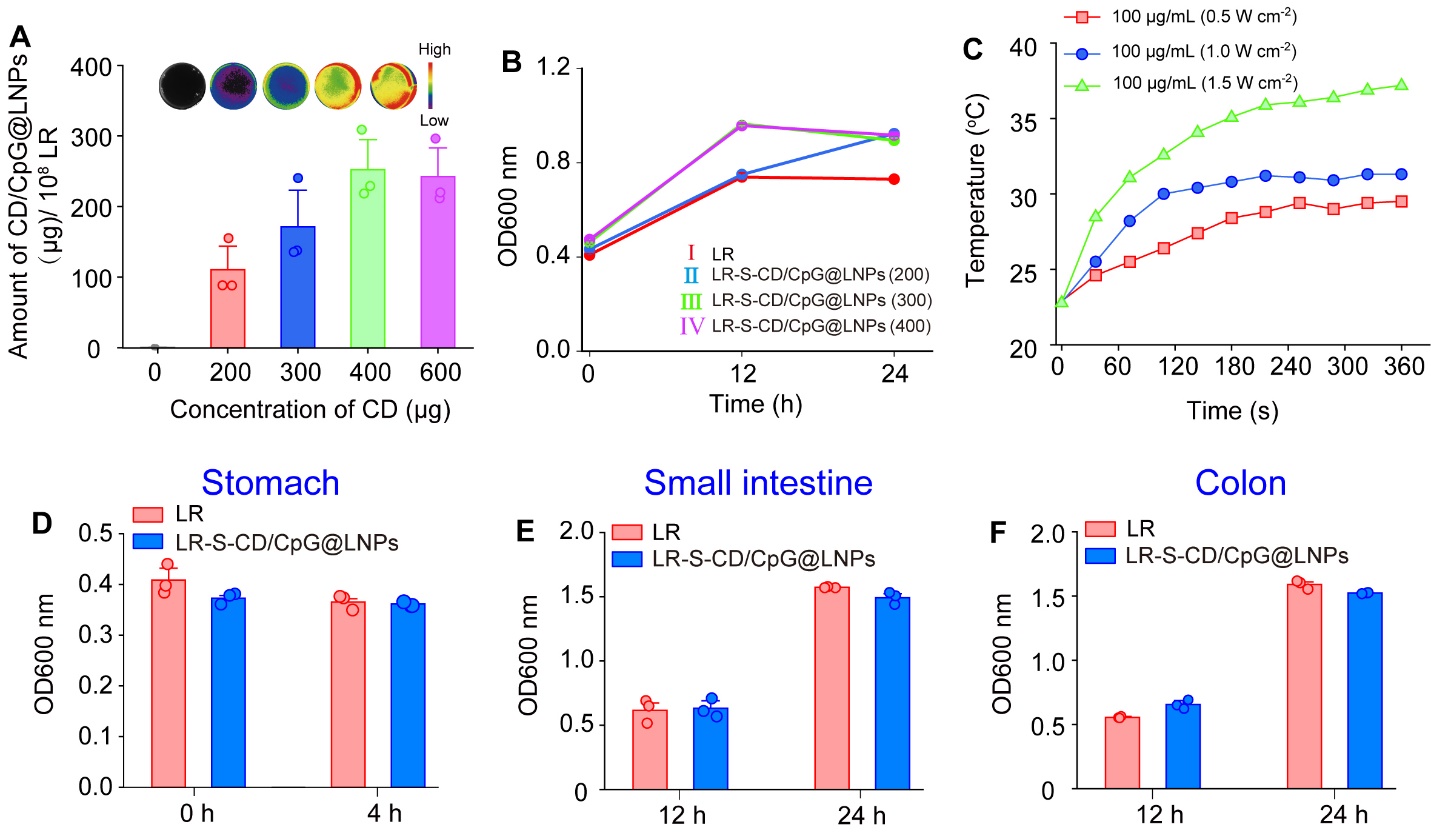


**Figure S5.** (A) The attached contents of CD/CpG@LNPs to 10^8^ CFU LR. (B) Growth curves of native and coated LR. Bacteria were cultured in an MRS medium at 37 ^o^C, and OD600 was measured at predetermined time points. (C) Temperature variations of LR-S-CD/CpG@LNP suspensions (100 μg mL^-1^) after NIR irradiation at different power for 360 s. Bacterial counts of LR and LR-S-CD/CpG@LNPs exposed to (D) simulated gastric fluid (pH 2.0), (E) simulated small intestine fluid (pH 6.8), and (F) simulated colonic fluid (pH 6.0), respectively (n = 3 independent experiments; Data are expressed as means ± s.e.m).


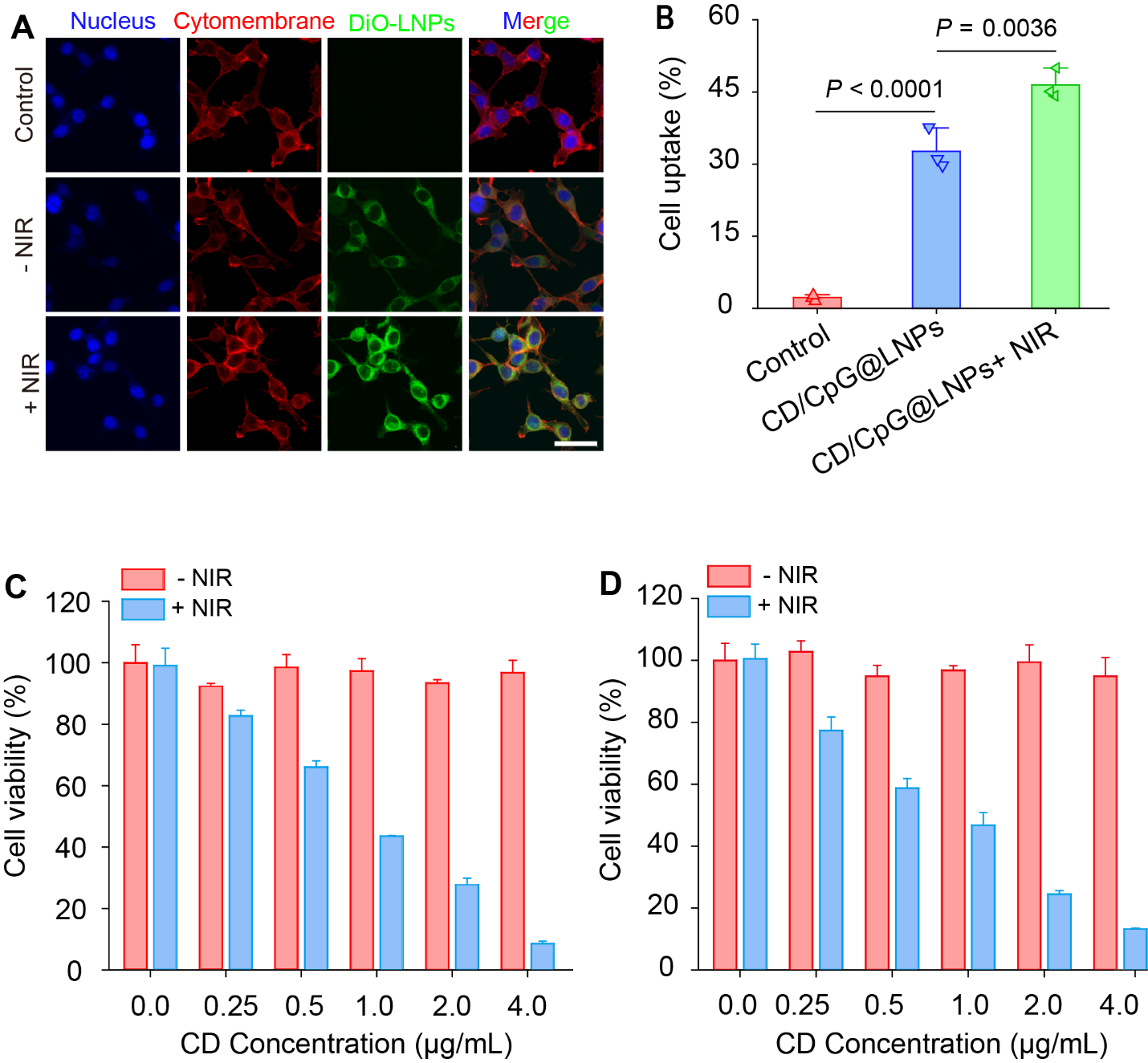


**Figure S6.** (A) CLSM images showing the internalization profiles of DiO-LNPs (DiO, 0.5 μM mL^-1^) by CT-26 cells after co-incubation for 4 h with or without 660 irradiation (0.5 W cm^-2^, 3 min). The scale bar represents 20 μm. (B) Cellular uptake percentages of CD/CpG@LNPs by CT-26 cells after co-incubation for 4 h with or without 660 irradiation (0.5 W cm^-2^, 3 min) (n = 3 independent experiments; **P* < 0.05, ***P* < 0.01, ****P* < 0.001, and *****P* < 0.0001 by one-way ANOVA with Tukey’s multiple comparison test). Viability of CT-26 cells after co-incubation with (C) CD@LNPs and (D) CD/CpG@LNPs for 24 h with or without 660 irradiation (0.5 W cm^-2^, 3 min) (n = 4 independent experiments; **P* < 0.05, ***P* < 0.01, ****P* < 0.001, and *****P* < 0.0001 by one-way ANOVA with Tukey’s multiple comparison test).


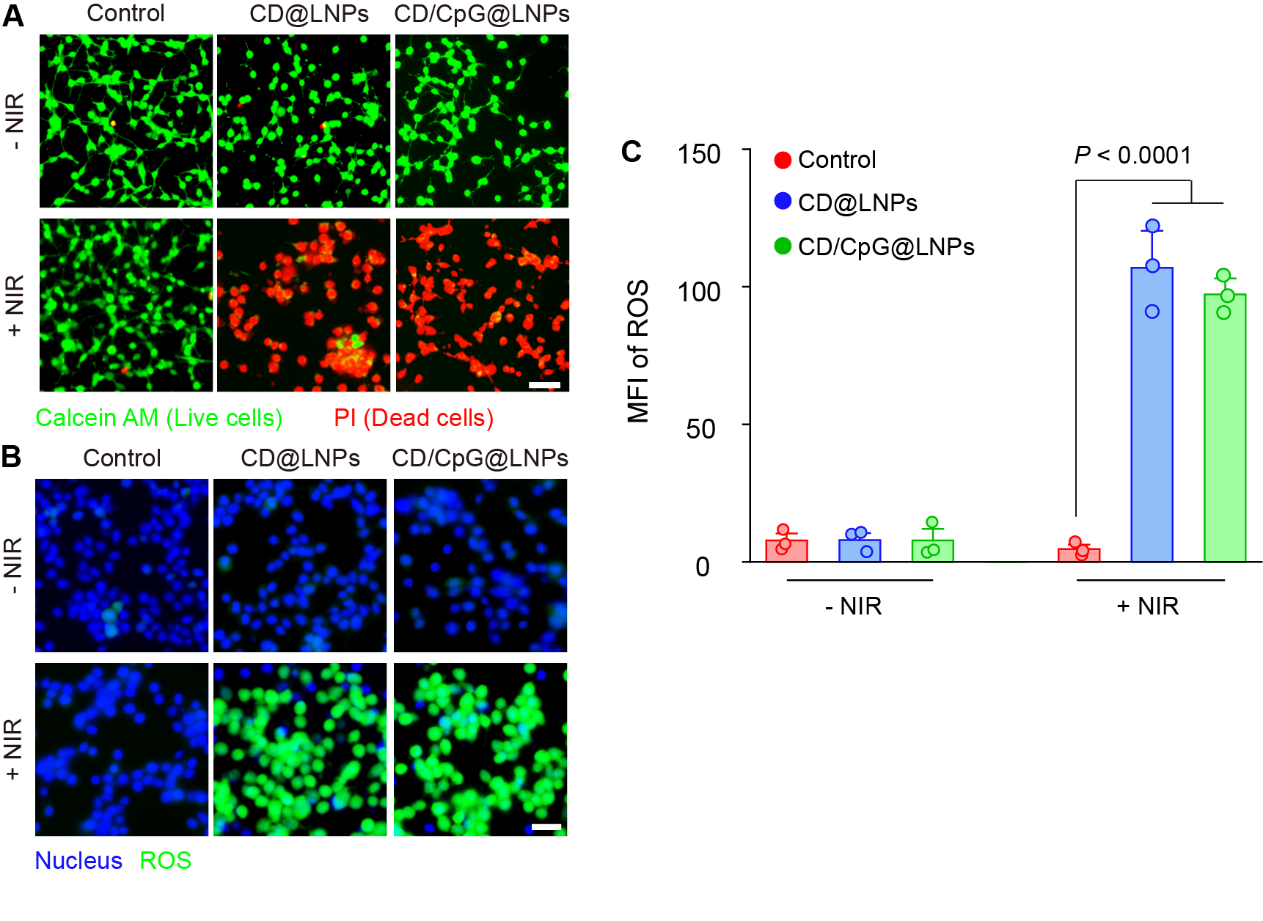


**Figure S7.** (A) Live/dead cell staining and (B) intracellular ROS detection of CT-26 cells after various treatments. (C) Quantification of the fluorescence intensities of intracellular ROS probe (n = 3 independent experiments; **P* < 0.05, ***P* < 0.01, ****P* < 0.001, and *****P* < 0.0001 by one-way ANOVA with Tukey’s multiple comparison test).


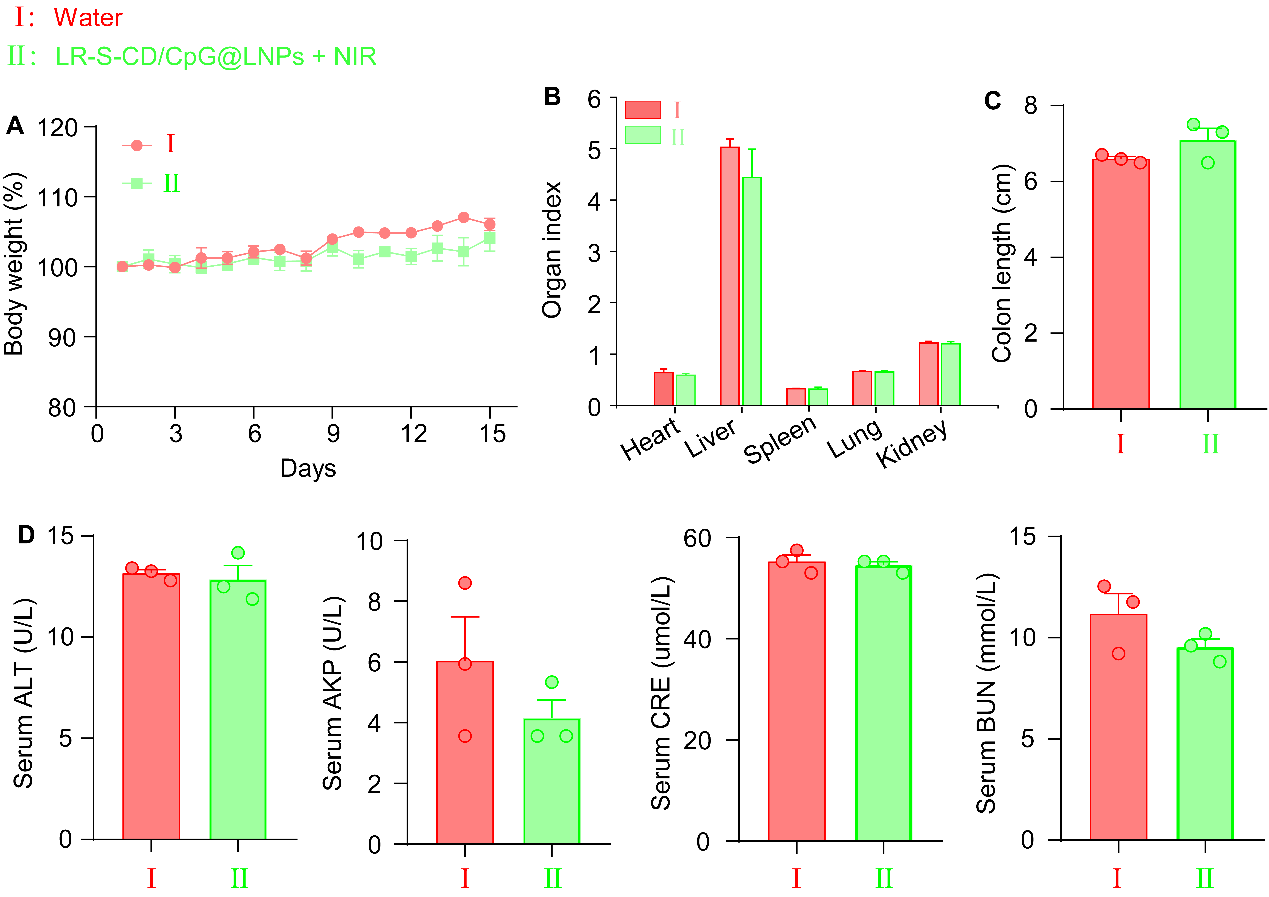


**Figure S8.** In vivo biosafety evaluation of LR-S-CD/CpG@LNPs. (A) Mouse body weight variations during the treatment period. (B) Organ indices, (C) colon lengths, and (D) serum biochemistry analysis of mice after various treatments (n = 3 biologically independent samples) on day 15.


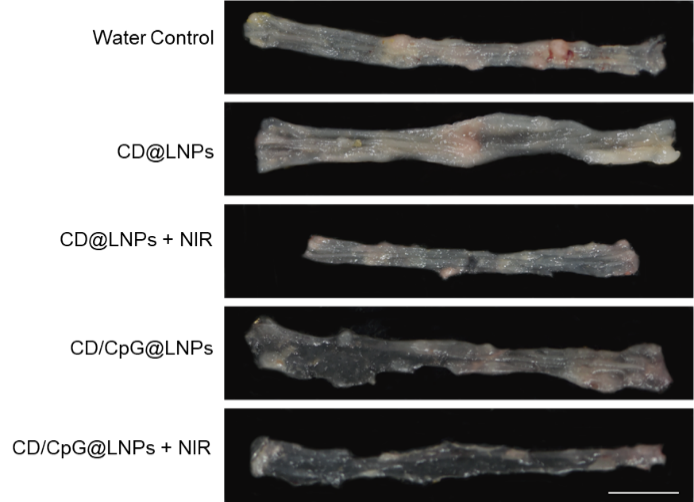


**Figure S9.** Images of colorectal tumors from various treatment groups at the end of treatments (Scale bar = 1 cm).

**
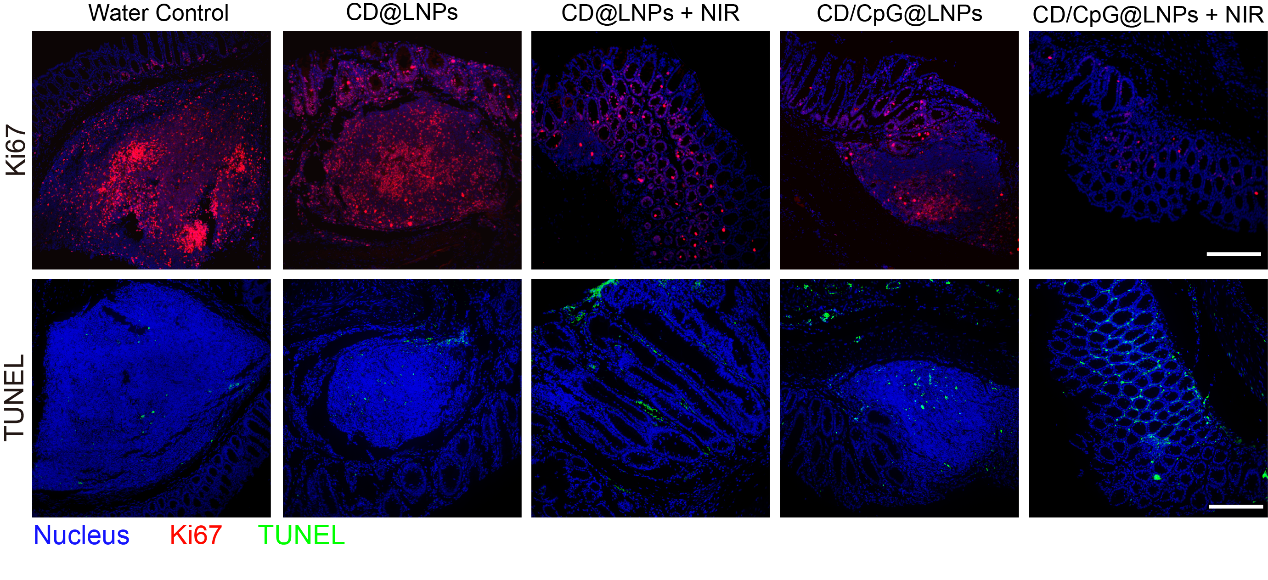
**

**Figure S10.** Ki67 and TUNEL staining of colorectal tumor sections from various mouse groups (scale bar = 100 μm).


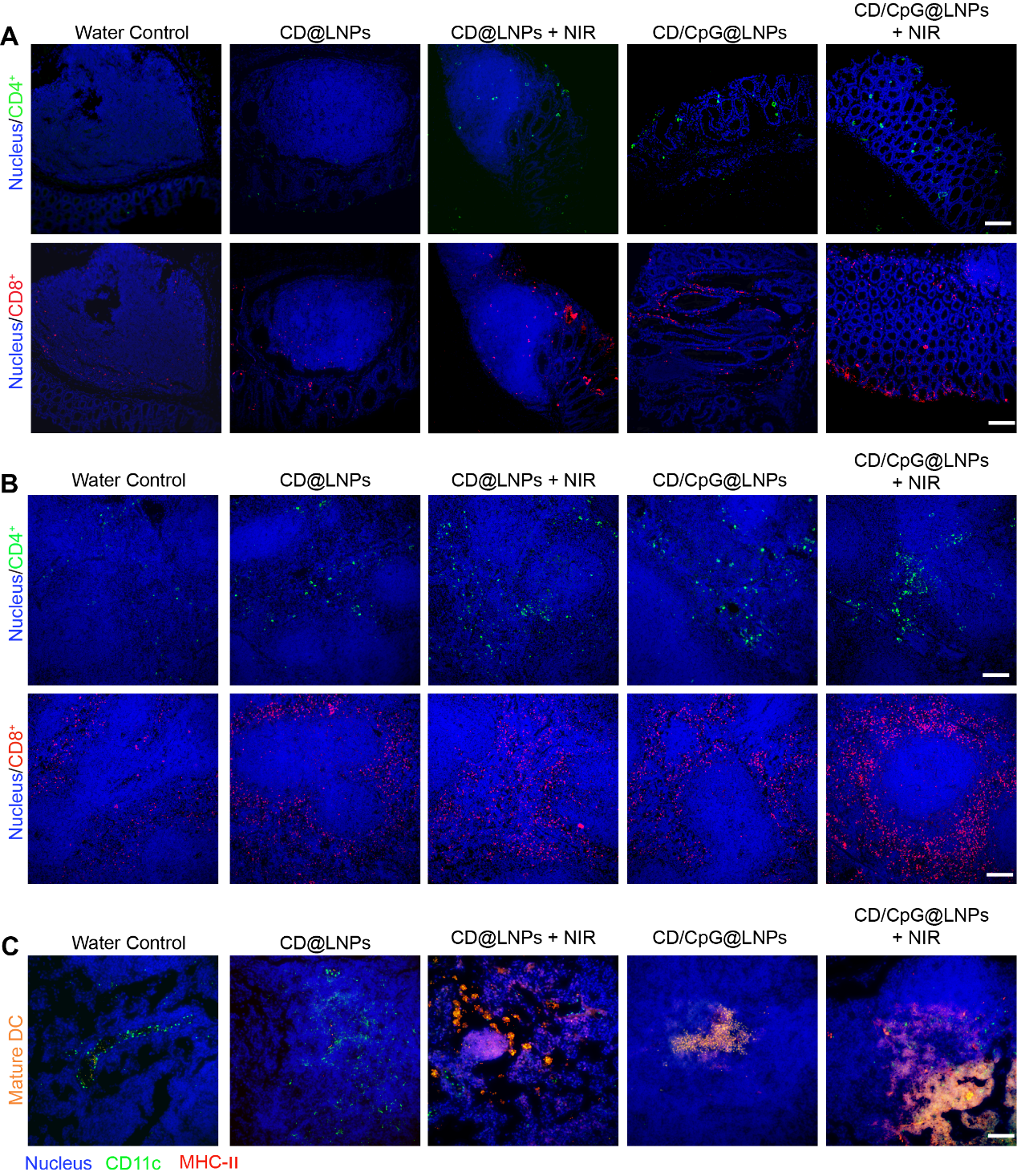


**Figure S11.** (A) CLSM images of colorectal tumor tissues after immunofluorescence staining (Red: CD8^+^ T cells; Green: CD4^+^ T cells). (B) CLSM images of spleen tissues after immunofluorescence staining (Red: CD8^+^ T cells; Green: CD4^+^ T cells). (C) CLSM images of lymph node tissues after immunofluorescence staining (Red: MHC-Ⅱ; Green: CD11c; Yellow: Mature DC; Scale bar = 100 μm).


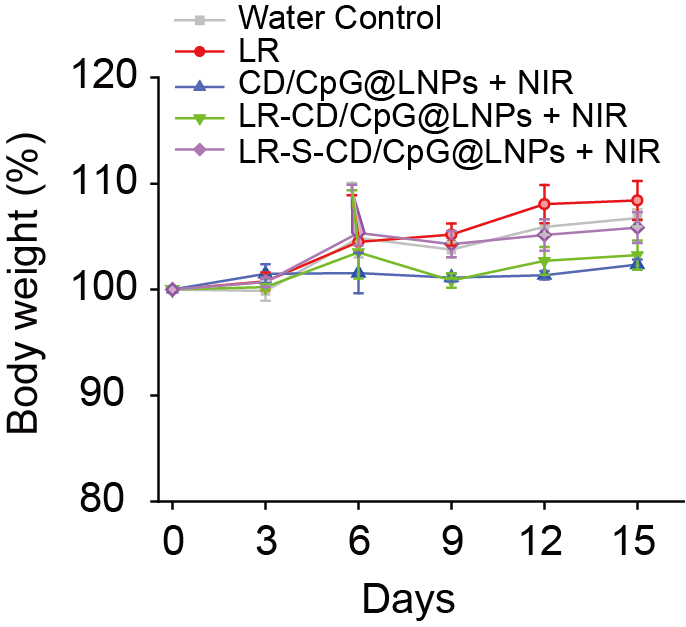


**Figure S12.** Body weight variations of different mouse groups during the treatment of orthotopic CRC (n = 5 biologically independent experiments; Data are expressed as means ± s.e.m.).


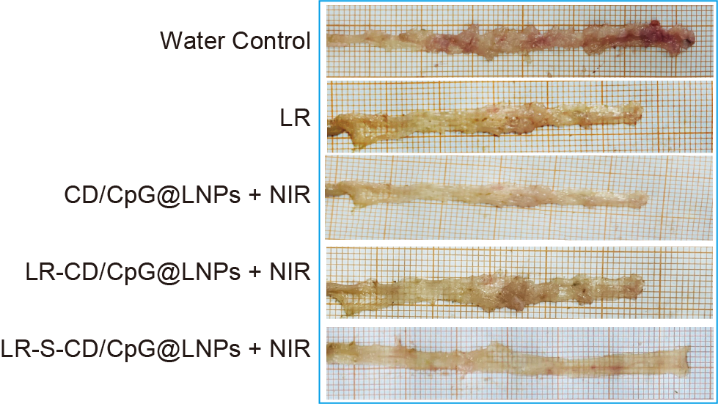


**Figure S13.** Images of colorectal tumors from various mouse groups.


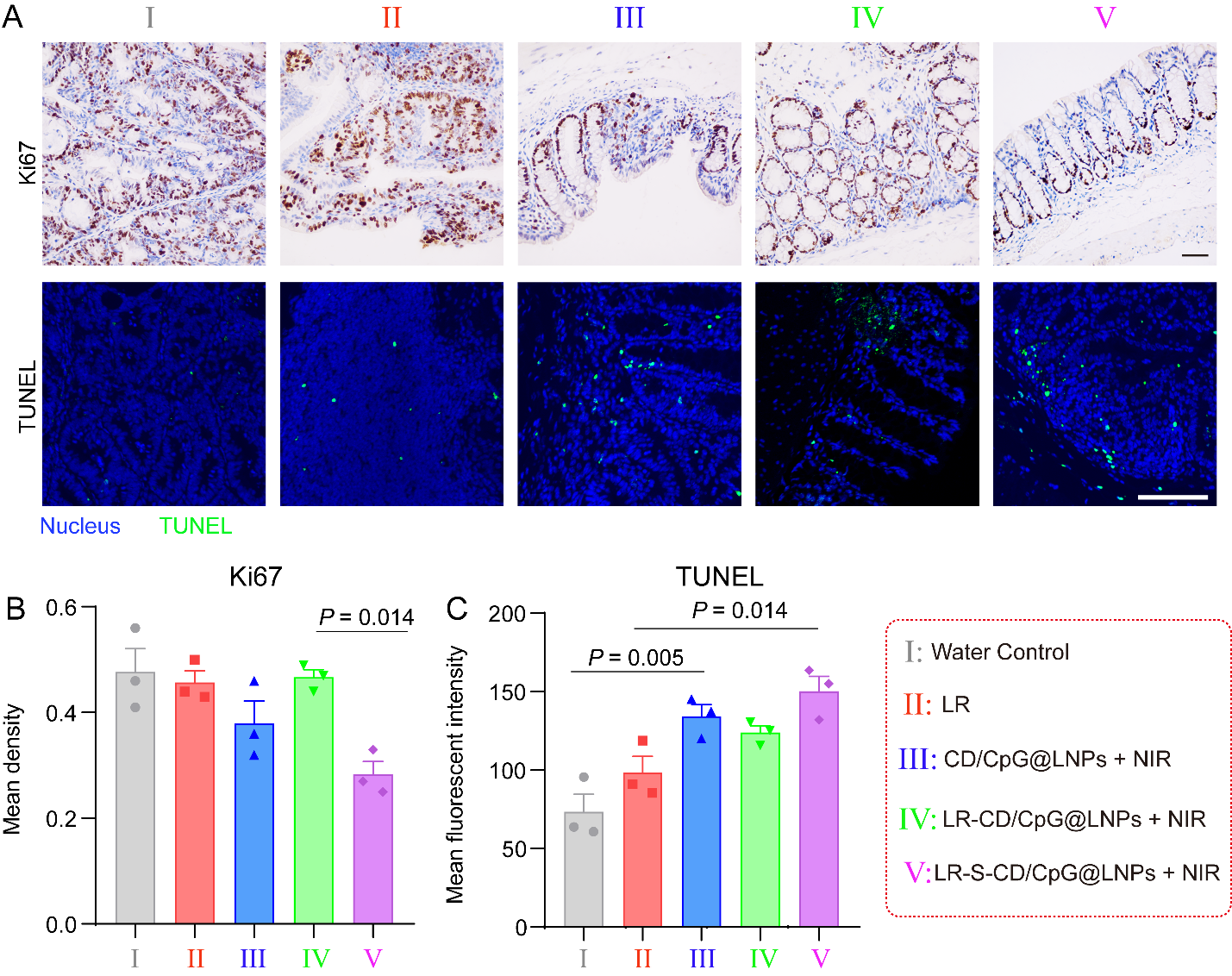


**Figure S14.** (A) Ki67 and TUNEL staining of colorectal tumor sections from various mouse groups (scale bar = 100 μm). Quantitative results of (B) Ki67-positive cells and (C) TUNEL-positive cells in colorectal tumor tissues from various treatment groups. Data are expressed as means ± s.e.m. (n = 3 biologically independent experiments; **P* < 0.05, ***P* < 0.01, ****P* < 0.001, and *****P* < 0.0001 by one-way ANOVA with Tukey’s multiple comparison test).


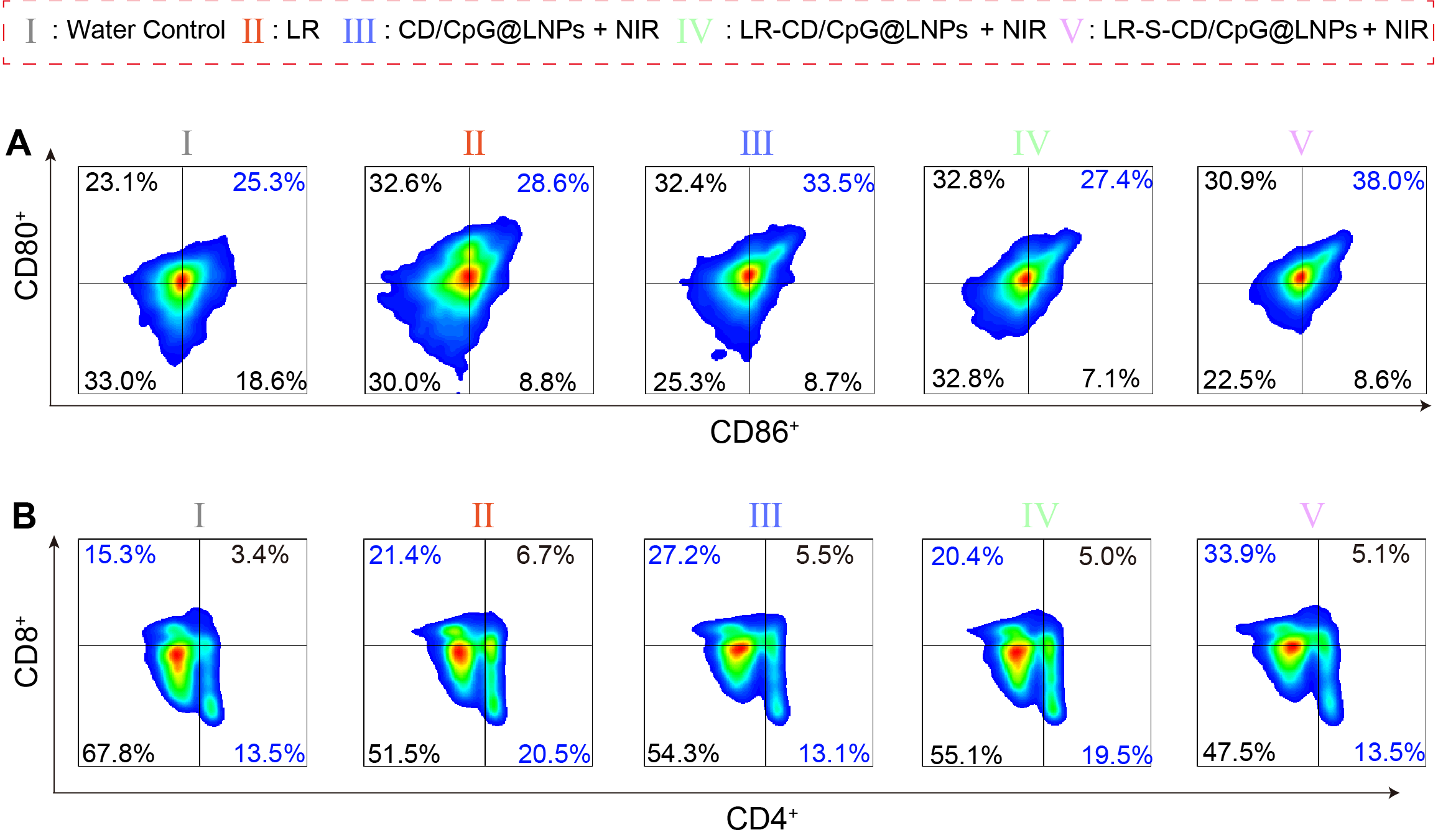


**Figure S15.** (A) FCM analysis of CD80^+^CD86^+^ in CD11c^+^ DCs from the tumor-draining lymph nodes. Cells were collected, stained, and assessed by FCM. (B) FCM analysis of CD8^+^ and CD4^+^ T cells in the spleens from various treatment groups.


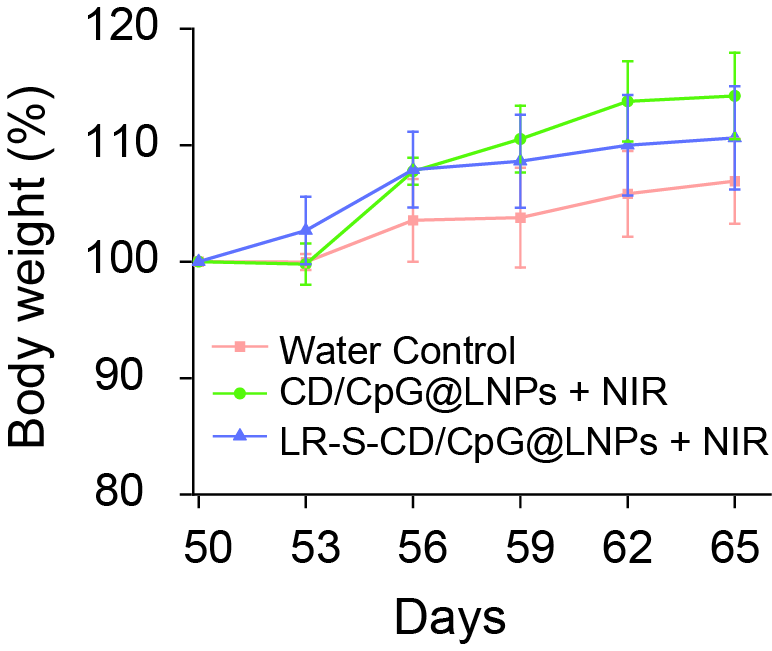


**Figure S16.** Body weights of mice in different groups throughout the treatment period (n = 4 biologically independent experiments; Data are expressed as means ± s.e.m.).


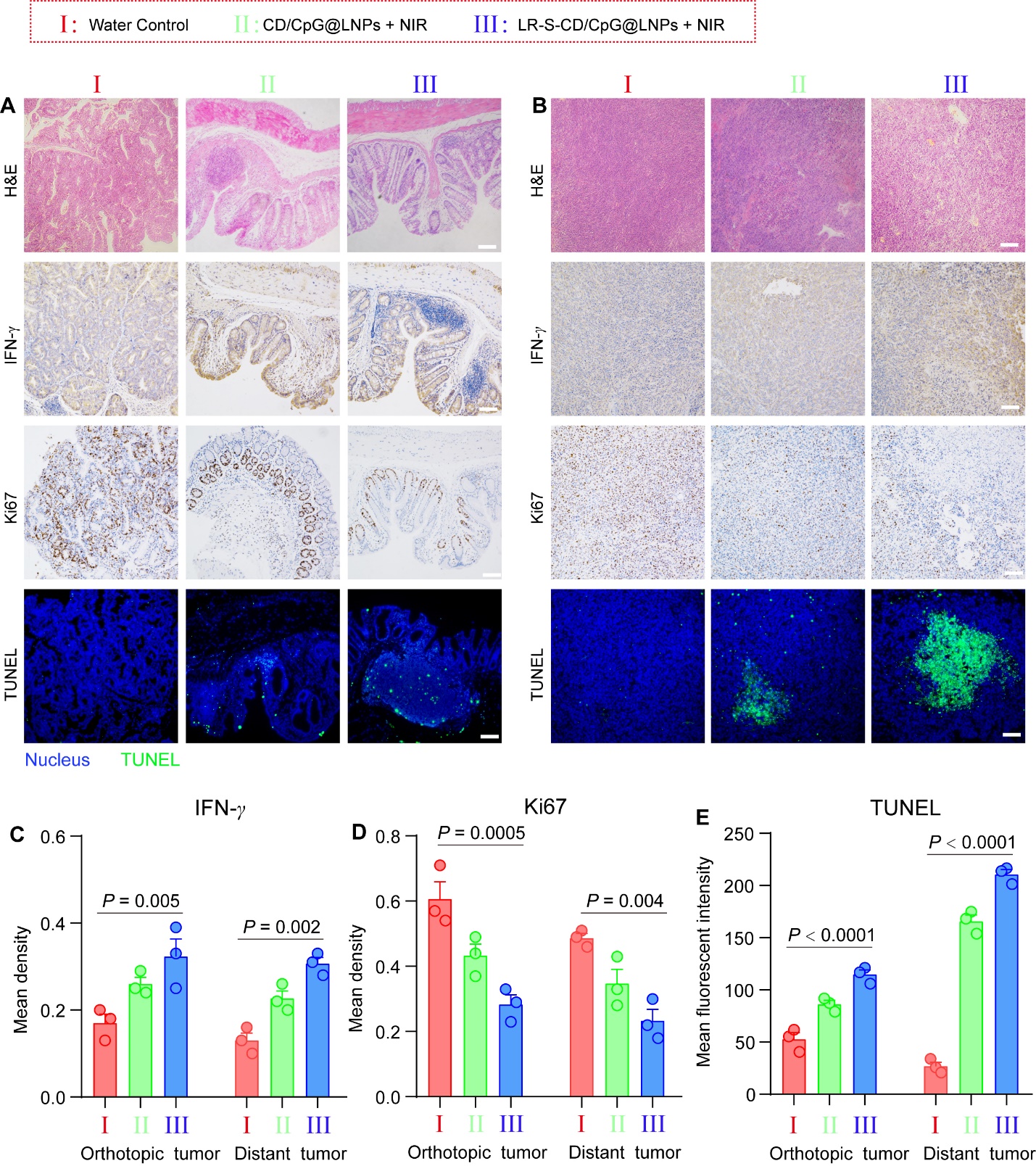


**Figure S17.** H&E, IFN-*γ*, Ki-67, and TUNEL staining of (A) colorectal tumors and (B) distant liver tumors following various treatments (scale bar: 100 μm). Quantitative results of (C) IFN-*γ*-positive cells, (D) Ki67-positive cells, and (E) TUNEL-positive cells in the colorectal tumors and distant liver tumor tissues. Data are expressed as means ± s.e.m. (n = 3 biologically independent experiments; **P* < 0.05, ***P* < 0.01, ****P* < 0.001, and *****P* < 0.0001 by one-way ANOVA with Tukey’s multiple comparison test).


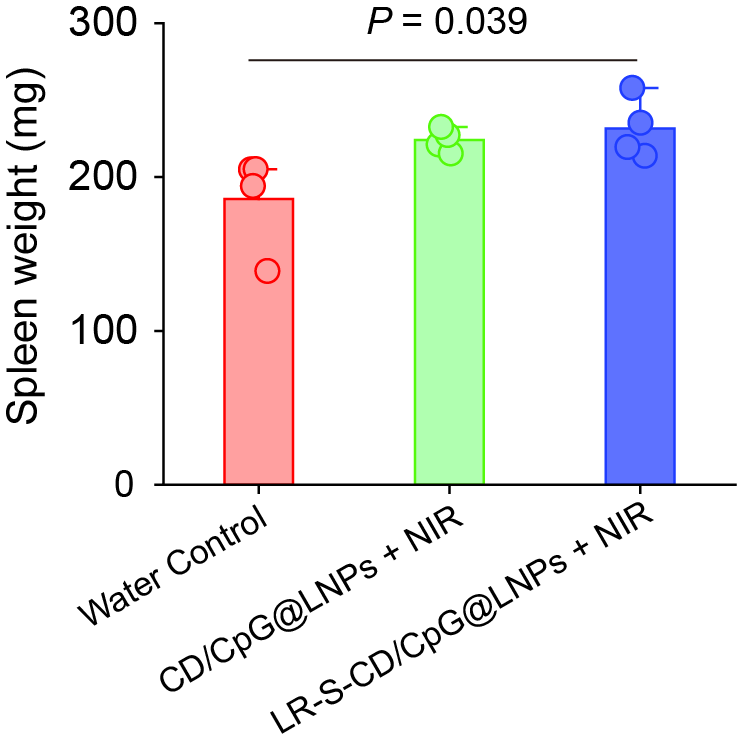


**Figure S18.** Spleen weights of mice in different groups at the end of treatments (n = 4 biologically independent experiments; **P* < 0.05, ***P* < 0.01, ****P* < 0.001, and *****P* < 0.0001 by one-way ANOVA with Tukey’s multiple comparison test).


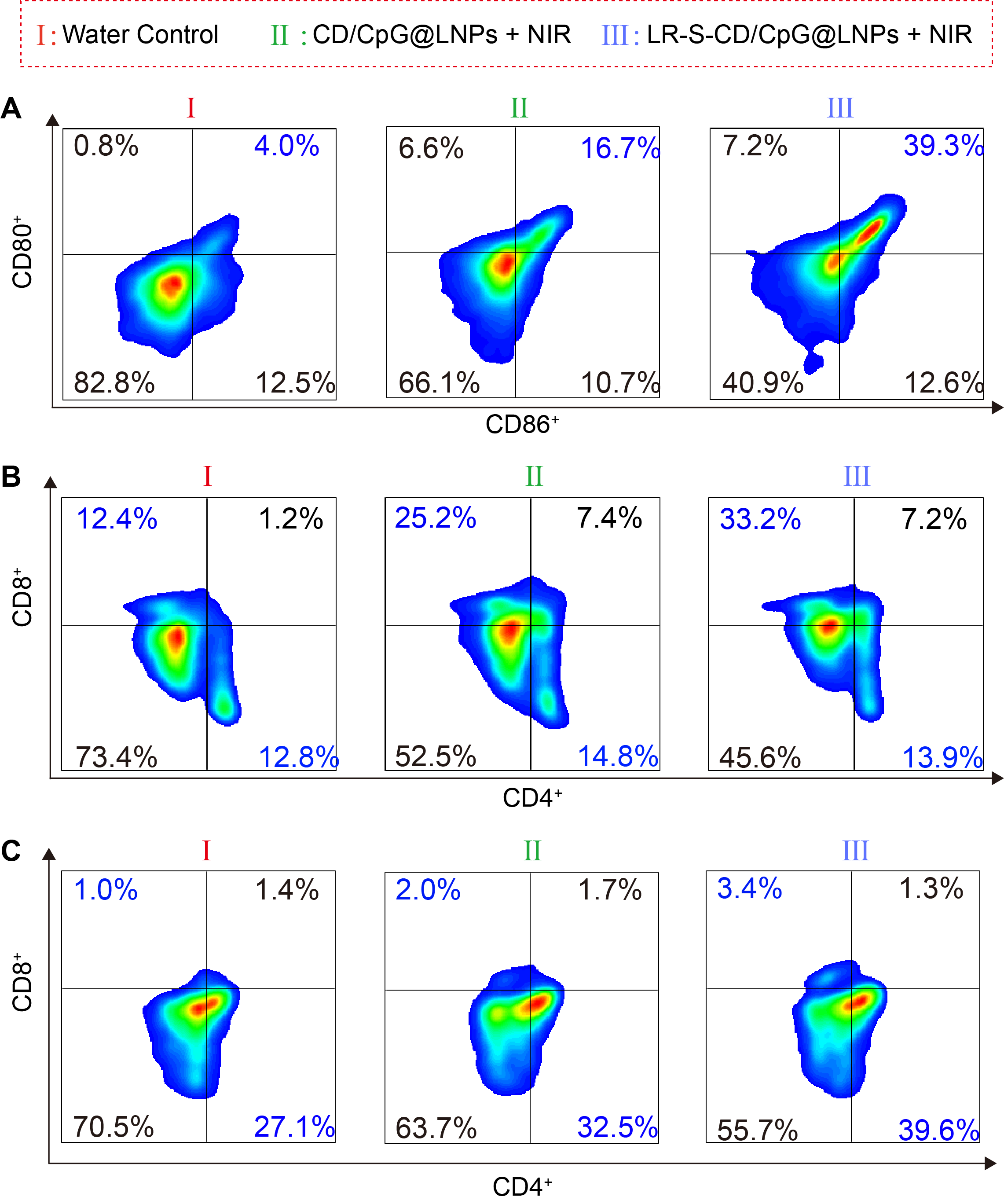


**Figure S19.** (A) FCM analysis of CD80^+^CD86^+^ in CD11c^+^ DCs from the tumor-draining lymph nodes. (B) FCM analysis of CD8^+^ and CD4^+^ T cells in the spleens from various treatment groups. (C) FCM analysis of CD8^+^ and CD4^+^ T cells in the distant tumor from various treatment groups.


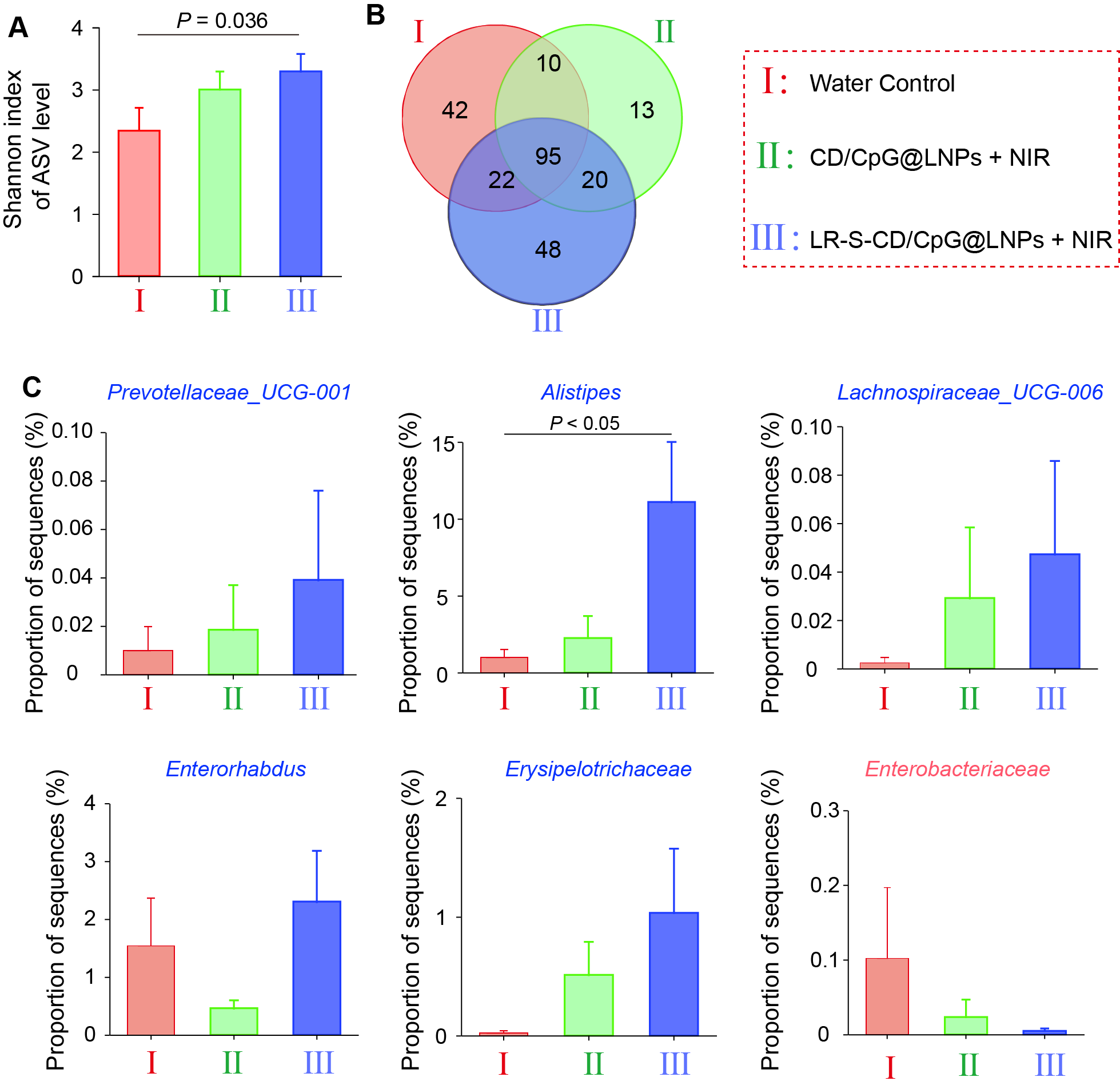


**Figure S20.** (A) Shannon indices on the ASV levels of microbes from various mouse groups. (B) Venn diagrams showing the richness of shared and unique bacterial species from various mouse groups. (C) Relative abundance of gut microbiota community members among various groups (n = 4 biologically independent experiments; **P* < 0.05, ***P* < 0.01, ****P* < 0.001, and *****P* < 0.0001 by one-way ANOVA with Tukey’s multiple comparison test).


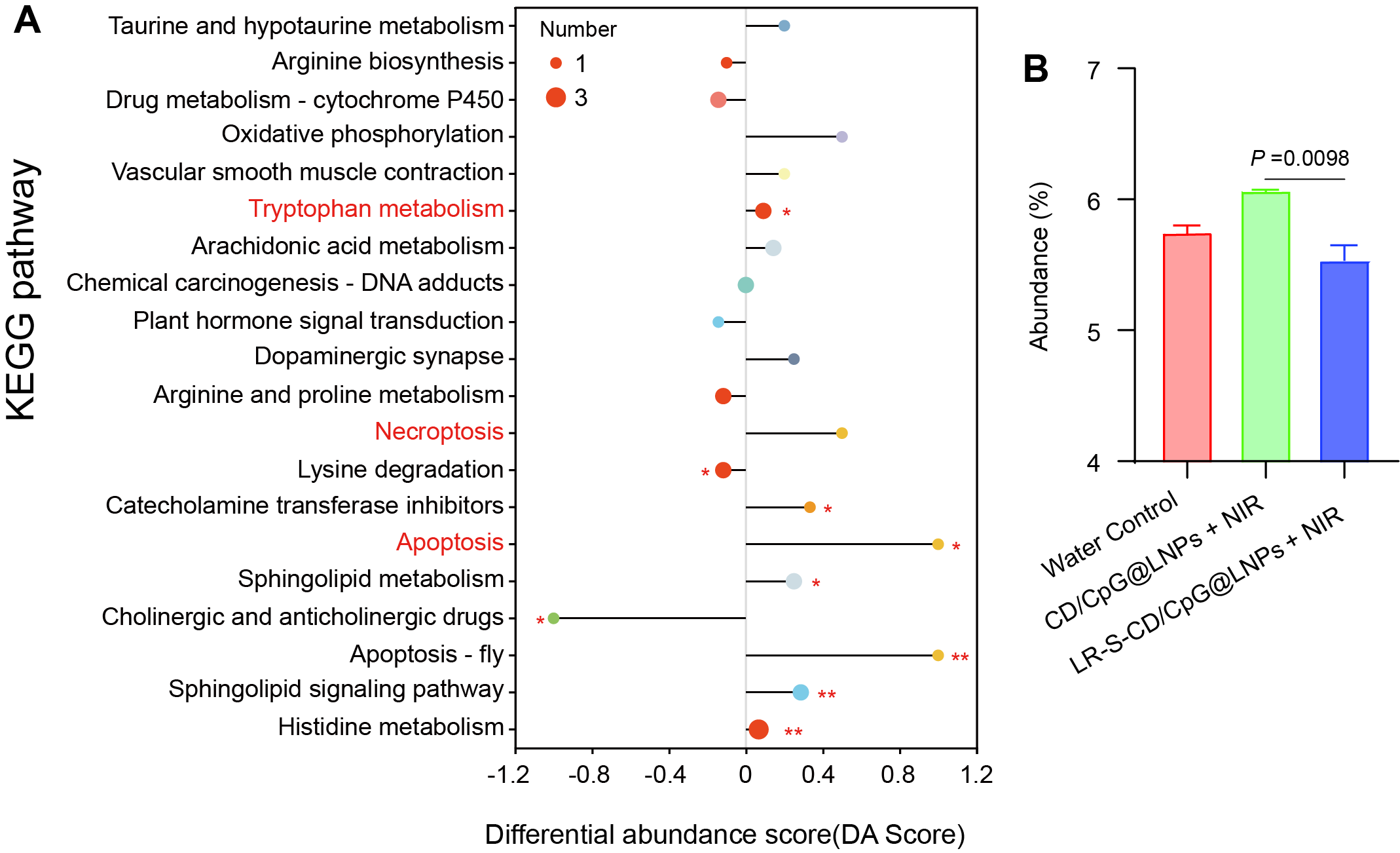


**Figure S21.** (A) Identified metabolites classified into the top 20 KEGG pathways. (B) The levels of tryptophan among different groups (n = 4 biologically independent experiments; **P* < 0.05, ***P* < 0.01, ****P* < 0.001, and *****P* < 0.0001 by one-way ANOVA with Tukey’s multiple comparison test).

**Table S1.** Hematological parameter analysis of the LR-S-CD/CpG@LNP-treated mice.

| Parameters | Water | LR-S-CD/CpG@LNPs |
| --- | --- | --- |
| WBC (× 10^9^/L) | 7.3 ± 1.1 | 6.7 ± 1.8 |
| Lym (× 10^9^/L) | 5.7 ± 0.8 | 5.3 ± 1.4 |
| Mon (× 10^9^/L) | 0.1 ± 0.06 | 0.2 ± 0.06 |
| Gran (× 10^9^/L) | 1.4 ± 0.4 | 1.2 ± 0.4 |
| RBC (× 10^12^/L) | 10.4 ± 0.2 | 8.9 ± 2.4 |
| HGB (g/L) | 154.0 ± 2.0 | 135.0 ± 38.0 |
| HCT (%) | 48.9 ± 0.3 | 41.8 ± 11.7 |
| MCV (fL) | 47.2 ± 0.4 | 46.7 ± 1.2 |
| MCH (pg) | 14.8 ± 0.1 | 15.0 ± 0.4 |
| MCHC (g/L) | 314.3 ± 2.5 | 322.0 ± 6.6 |
| RDW (%) | 15.6 ± 0.5 | 15.4 ± 0.4 |
| PLT (× 10^9^/L) | 1165.3 ± 117.7 | 1146.0 ± 333.6 |
| MPV (fL) | 4.5 ± 0.3 | 4.6 ± 0.2 |
| PDW | 16.1 ± 0.1 | 16.0 ± 0.2 |
| PCT (%) | 0.4 ± 0.1 | 0.5 ± 0.2 |

WBC, White blood cell count; Lym, Lymphocytes; Mon, Monocytes; Gran, Granulocyte; RBC, Red blood cell counts; HGB, Hemoglobin; HCT, Hematocrit; MCV, Mean corpuscular volume; MCH, Mean corpuscular hemoglobin; MCHC, Mean corpuscular hemoglobin concentration; RDW, Red cell distribution width; PLT, Platelets; MPV, Mean platelet volume; PDW, Platelet distribution width; PCT, Plateletcrit. Data are presented as mean ± s.e.m. (n = 3 biologically independent experiments).

**References**

[1] J. Liu, T. Kong, H. M. Xiong, Mulberry-leaves-derived red-emissive carbon dots for feeding silkworms to produce brightly fluorescent silk. Adv. Mater. 34 (2022) e2200152, https://doi.org/10.1002/adma.202200152.
